# The flavonoid of *Dracocephalum heterophyllum* Benth. ameliorates cerebral small vessel disease by inhibiting the autophagy via Angs-Tie2 signaling pathway

**DOI:** 10.3389/fphar.2025.1500307

**Published:** 2025-05-13

**Authors:** Lin Liu, Wen He, Sijing Liu, Yang Li, Peng Wang, Fang Yan, Wenjing Yang, Yongxue Yang, Jinlin Guo

**Affiliations:** ^1^ College of Pharmacy, Chengdu University of Traditional Chinese Medicine, Chengdu, China; ^2^ Geriatric Diseases Institute of Chengdu/Cancer Prevention and Treatment Institute of Chengdu, Department of Clinical Trial Center, Chengdu Fifth People’s Hospital (The Second Clinical Medical College, Affiliated Fifth People’s Hospital of Chengdu University of Traditional Chinese Medicine), Chengdu, China; ^3^ College of Medical Technology, Chengdu University of Traditional Chinese Medicine, Chengdu, China; ^4^ College of Public Health, Chengdu University of Traditional Chinese Medicine, Chengdu, China

**Keywords:** flavonoid of Dracocephalum heterophyllum Benth., human umbilical vein endothelial cells, cerebral small vessel disease, Angiogenin, autophagy

## Abstract

**Background:**

Cerebral small vessel disease (CSVD) is a common cause of stroke and vascular cognitive impairment. It is urgent to find drugs targeting CSVD. This study explores the therapeutic potential of the flavonoid (DHBF) derived from *Dracocephalum heterophyllum* Benth., a traditional Tibetan medicine used for cardiovascular diseases, in treating CSVD and its underlying mechanisms.

**Methods:**

Spontaneously hypertensive rats (24-weeks-old) were treated with DHBF for 8 weeks. The Morris water maze test, laser speckle contrast imaging, photoacoustic tomography, HE and Nissl staining were used to evaluate the effect of DHBF in CSVD rats. Network pharmacology and UPLC-MS were used to identify DHBF components and potential mechanisms. Human umbilical vein endothelial cells (HUVECs) were exposed to 10% O_2_ to mimic CSVD conditions, and the effects of DHBF on proliferation, migration, and autophagy were evaluated. The expression levels of Angs, Tie2, LC3-Ⅱ/Ⅰ and p62 were detected by qRT-PCR and WB analyses. Molecular docking and lentivirus-mediated Ang2 knockdown/overexpression were performed to validate DHBF’s targeting of Ang2.

**Results:**

DHBF alleviated vessel injury, improved learning and memory abilities, and increased cerebral blood perfusion and oxygen supply capacity in 24-week-old CSVD rats (*p* < 0.05). A total of 31 components of DHBF were identified by UPLC-Q-Orbitrap HRMS. Results indicated that DHBF alleviated CSVD by promoting cell proliferation, migration and invasion while inhibiting autophagy in endothelial cell. This regulation was associated with alterations in the Angs-Tie2 pathway and its downstream proteins, including decreased levels of Ang2, Tie2, LC3-Ⅱ/LC3-Ⅰ and increased the levels of Ang1 and p62 (*p* < 0.05). Knocking down Ang2 showed regulatory effects similar to those observed with DHBF intervention, while overexpression of Ang2 showed opposite effects. In addition, Ang2 overexpression attenuated the regulatory effects of DHBF on Angs-Tie2 pathways and autophagy in HUVECs. These results demonstrated that DHBF alleviated CSVD via inhibiting Ang2, which might be related to danshensu, lonicerin, 8-hydroxyquinoline, esculetin, isophorone, and ethyl caffeate.

**Conclusion:**

In summary, DHBF exerts a therapeutic effect on CSVD by inhibiting Ang2, regulating the Angs-Tie2 pathway, and inhibiting endothelial autophagy. This study proposed a potential effective target for CSVD, provided data to support subsequent drug development for this condition.

## Graphical Abstract

**Figure:**
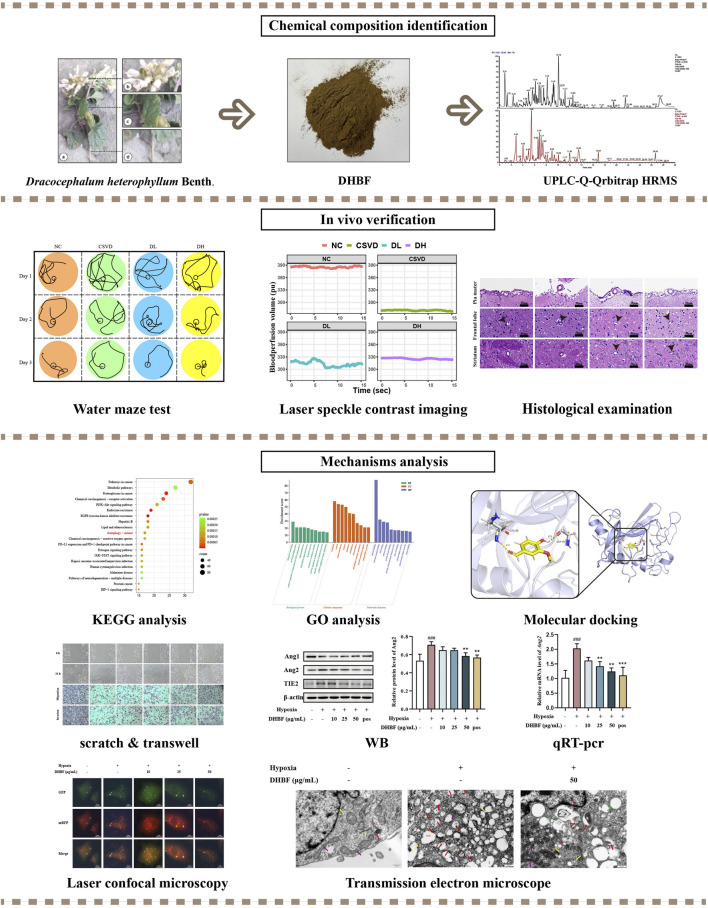

## 1 Introduction

Cerebral small vessel disease (CSVD) encompasses a range of diseases that damage the cerebral vascular system including small arteries, arterioles, capillaries, and venules (Pantoni, 2010). CSVD is a common cause of stroke and represents the most prevalent pathological basis for vascular cognitive impairment (VCI) (Rost et al., 2022; Wardlaw et al., 2023). It is estimated that approximately 25% of ischemic strokes and the majority of intracerebral hemorrhages are associated with CSVD (Parodi et al., 2023). The VCI caused by CSVD accounts for at least 45% of dementia cases worldwide and significantly affects the quality of life of patients (Wardlaw et al., 2022). Rather than being a singular condition, CSVD is a systemic disease that can arise from various pathological processes. One of the most common types of CSVD is arteriosclerosis (Litak et al., 2020), which is mainly characterized by the accumulation of fibrous transparent material, luminal stenosis, and remodeling of vascular wall (Pantoni, 2010). Hypertension is the primary risk factor for CSVD, resulting in media lipohyalinosis, vascular wall remodeling, and luminal narrowing (Liu et al., 2018). Consequently, the clinical application of lipid-lowering and antihypertensive drugs may be a potential treatment (Xu et al., 2021). Despite ongoing research into stroke and VCI in patients with CSVD, specific treatments for CSVD remain lacking.

*Dracocephalum heterophyllum* Benth. belongs to the Labiatae family and is broadly distributed in Tibet, Qinghai, Xinjiang, Gansu, Western Sichuan, Inner Mongolia, and Shanxi Province in China (Dang et al., 2018). As a traditional Tibetan medicine, this herb is used to treat hypertension, as documented in many books, such as *Guide of Common Chinese Herb Medicine in Qinghai*, *Drug pictorial Manual of Qinghai-Xizang Plateau*, *Xinjiang Medicinal Plant Journal*, *National Compendium of Chinese Herbal Medicine* and *Chinese Materia Medica*. Our previous studies have proved that the flavonoid of *D. heterophyllum* Benth. (DHBF) could reduce blood pressure, protect vascular endothelium (He, 2013b), and inhibit cardiac hypertrophy in hypertensive rats (He, 2013a). Some studies also showed that *D. heterophyllum* Benth. improved the anti-hypoxia ability (Peng, 1984) and protected the ultrastructure of the hippocampus (Xie et al., 1998) and cerebral cortex (Ma et al., 1996) in hypoxic rats. Furthermore, it has been reported that the DHBF have protective properties against cerebral ischemia reperfusion injury (Jia et al., 2017), as well as ameliorating the symptoms of chronic mountain sickness (Abudouwayiti et al., 2021). Therefore, DHBF may have a function in ameliorating CSVD, but related studies are still lacking.

Angiopoietins (Angs) are essential in the development of cardiovascular and cerebrovascular diseases. In particular, levels of Angiopoietin-2 (Ang2) are dramatically elevated in cardiovascular and cerebrovascular diseases, such as coronary heart disease (Wu et al., 2016) and stroke (Liu et al., 2009), with disease severity positively correlated with increased Ang2 levels (Chen et al., 2020; Peplinski et al., 2021). Research has demonstrated a significant association between Ang2 and white matter hyperintensity scores of CSVD as observed in brain magnetic resonance imaging (Bijkerk et al., 2022). Additionally, the Angs-Tie2 pathway plays a major role in mediating angiogenesis. Ang1 activates Tie2 to maintain vascular stability and promote vessel maturation, while Ang2 leads to vascular degeneration, destabilization, and remodeling by inhibiting Tie2 (Zeng et al., 2016; Magkouta et al., 2019). Impaired autophagy has been reported to induce vascular endothelium morphological abnormalities and dysfunction in diabetes (Liu et al., 2023). Conversely, excessive autophagy induced by advanced glycation end products can also impair angiogenic dysfunction (Xiong et al., 2021). Furthermore, it has been proved that Angs could mediate autophagy (Yin et al., 2019). However, the specific role of autophagy in CSVD remains to be elucidated. Therefore, we hypothesized that DHBF might improve CSVD by regulating endothelial autophagy and inhibiting vascular destabilization through Angs.

Therefore, we aimed to determine whether DHBF alleviates CSVD, explore the major component of DHBF, and investigate the effect and underlying mechanisms of DHBF treating CSVD in hypoxic endothelial cells.

## 2 Methods

### 2.1 Extraction of DHBF

The plants were identified as *D. heterophyllum* Benth. by researcher Guan-Mian Sheng from the Xinjiang Technical Institute of Physics and Chemistry in the Chinese Academy of Sciences. DHBF (20200102) was produced by the pilot plant of Xinjiang Technical Institute of Physics and Chemistry Chinese Academy of Sciences. The details were described in Supplementary Material and methods.

### 2.2 UPLC-Q-Orbitrap HRMS analysis

The DHBF sample was analyzed using a UPLC-Q-Orbitrap HRMS system (Thermo Fisher Scientific, United States). Chromatographic separation was performed on an Accucord TM Vanquish C_18_ column (3 mm × 100 mm, 2.6 μm). The details were described in Supplementary Material and methods.

### 2.3 GO and KEGG enrichment analysis and protein-protein interaction (PPI) network analysis

GO and KEGG enrichment analyses were performed to predict the mechanisms involved in the alleviation of CSVD by DHBF. A PPI analysis was performed by submitting the intersection targets to STRING (https://cn.string-db.org/), which was used to screen the key targets of DHBF to improve CSVD. The details were described in the Supplementary Material and methods.

### 2.4 Animals

Thirty-six 6-week-old male spontaneously hypertensive (SHR) rats and twelve 6-week-old male Wistar-Kyoto (WKY) rats were obtained from Beijing Vital River Laboratory Animal Technology Co., Ltd., They were fed a standard diet until they reached 24 weeks of age. The SHR rats were randomly divided into 3 groups (n = 12 per group): the model group (CSVD), the low dose of DHBF group (DL), the high dose of DHBF group (DH) and WKY rats served as the normal control group (NC). The dosages of DL and DH groups were 300 mg/kg and 600 mg/kg, respectively, as referenced in a previous study (Wen et al., 2021). DHBF was suspended in distilled water for intragastric administration.

### 2.5 Morris Water Maze Test

After an 8-weeks drug intervention, a 4-day Morris water maze test was conducted. Adaptive training was performed on the first day (Day 0), followed by the spatial acquisition trial on the second to the fourth days (Day 1 – Day 3). The details were described in the Supplementary Material and methods.

### 2.6 Laser speckle contrast imaging

To evaluate the effects of DHBF on cerebral blood perfusion in rats, we used the laser speckle contrast imaging (LSCI) system. After anesthetizing the rats, skin incisions were made to expose the skull and the periosteum was removed. A high-speed dental drill was then used to uniformly thin a 20 × 20 mm^2^ cranial window of the cerebral cortex until the pial vasculature was visible. The laser probe was positioned approximately 10 cm above the cranial window for image acquisition, and the cerebral blood perfusion volume was quantitatively measured for 15 s.

### 2.7 Photoacoustic tomography

To evaluate the influence of DHBF on cerebral blood flow (CBF) and the oxygen supply capacity of the cerebral cortex, photoacoustic tomography (PAT) was performed after LSCI. The PAT imaging system has been described previously (Yang et al., 2019). The details were presented in Supplementary Material and methods.

### 2.8 Histological examination

To determine whether DHBF alleviates CSVD, rats were euthanized after PAT, and brain tissues including the prefrontal cortex, hippocampus, pia mater, frontal lobe, and striatum were harvested and fixed with 4% paraformaldehyde for preparation of paraffin tissue sections. All embedded tissues were cut into 5 μm thick slices. hematoxylin-eosin (HE) staining and Nissl staining were used to analyze the pathological lesions in the prefrontal cortex and hippocampal regions, including CA1 to CA4. The pia mater, frontal lobe, and striatum were stained with HE to observe the morphological changes of cerebral small arteries. The inner and outer diameters of the cerebral small arteries were recorded and their ratio was calculated.

### 2.9 Cell culture and treatment

According to the previous study, human umbilical vein endothelial cells (HUVECs) were used for our investigation (Zhou et al., 2019). HUVECs were purchased from Procell Life Science and Technology Co., Ltd. (China) and cultured in HUVEC-specific medium, which contained Ham’s F-12K, 0.1 mg/mL Heparin, 0.05 mg/mL ECGs, 10% FBS and 1% penicillin/streptomycin, and incubated in a 37°C, 5% CO_2_ incubator. For the control group, cells were collected after being cultured for 72 h under normal conditions. For the hypoxic group, after being incubated at the control conditions for 24 h, cells were exposed to hypoxia (10% O_2_, 5% CO_2_, 85% N_2_) in the incubator for 48 h. After hypoxic exposure, cells were treated with Nicorandil (0.1 mmol/L, Positive group) and various concentrations (10, 25, and 50 mg/mL) DHBF for 24 h, as referenced in a previous study (Hong, 2018).

### 2.10 Lentiviral Ang2 knockdown and overexpression

HUVECs were subjected to lentiviral transduction using lentiviral particles (shRNA Lentiviral Transduction; Shanghai Genechem Co., Ltd.), with a sequence targeting human Ang2. The human Ang2 lentiviral vector (Knockdown group and overexpression group) and the empty lentiviral vector (Ang2 vector group) were provided by Shanghai Genechem Co., Ltd. The viral co-infection reagent (HitransG P) was also obtained from Gene-chem. The lentivirus transduction efficacy was measured by microscope observation of fluorescence intensity.

### 2.11 Scratch wound healing assay and transwell assay

The proliferation, migration, and invasion ability were evaluated by scratch wound healing assay and transwell assay. The procedures were presented in the Supplementary Material and methods.

### 2.12 Quantitative real-time polymerase chain reaction (qRT-PCR) and Western blot (WB)

The mRNA and protein expression levels were detected by qRT-PCR and WB, the details were presented in the Supplementary Material and methods.

### 2.13 Laser scanning confocal microscopy (LSCM) and transmission electron microscope (TEM)

The autophagy levels were evaluated using LSCM and TEM, respectively. The details were described in the Supplementary Material and methods.

### 2.14 Molecular docking

The ingredients of DHBF identified by UPLC-Q-Orbitrap HRMS were chosen for molecular docking with Ang2. The specific process was presented in Supplementary Material and methods.

### 2.15 Statistics

All data were present as the mean ± standard deviation. SPSS (version 17) was used for data analysis. Analysis of variance (ANOVA) followed by LSD test was used for multiple comparisons between groups. The non-parametric test was performed by the Kruskal-Wallis test. *p* < 0.05 indicates a significant difference.

## 3 Results

### 3.1 DHBF alleviates arteriolosclerosis and improves the learning and memory ability of CSVD rats

**FIGURE 1:**
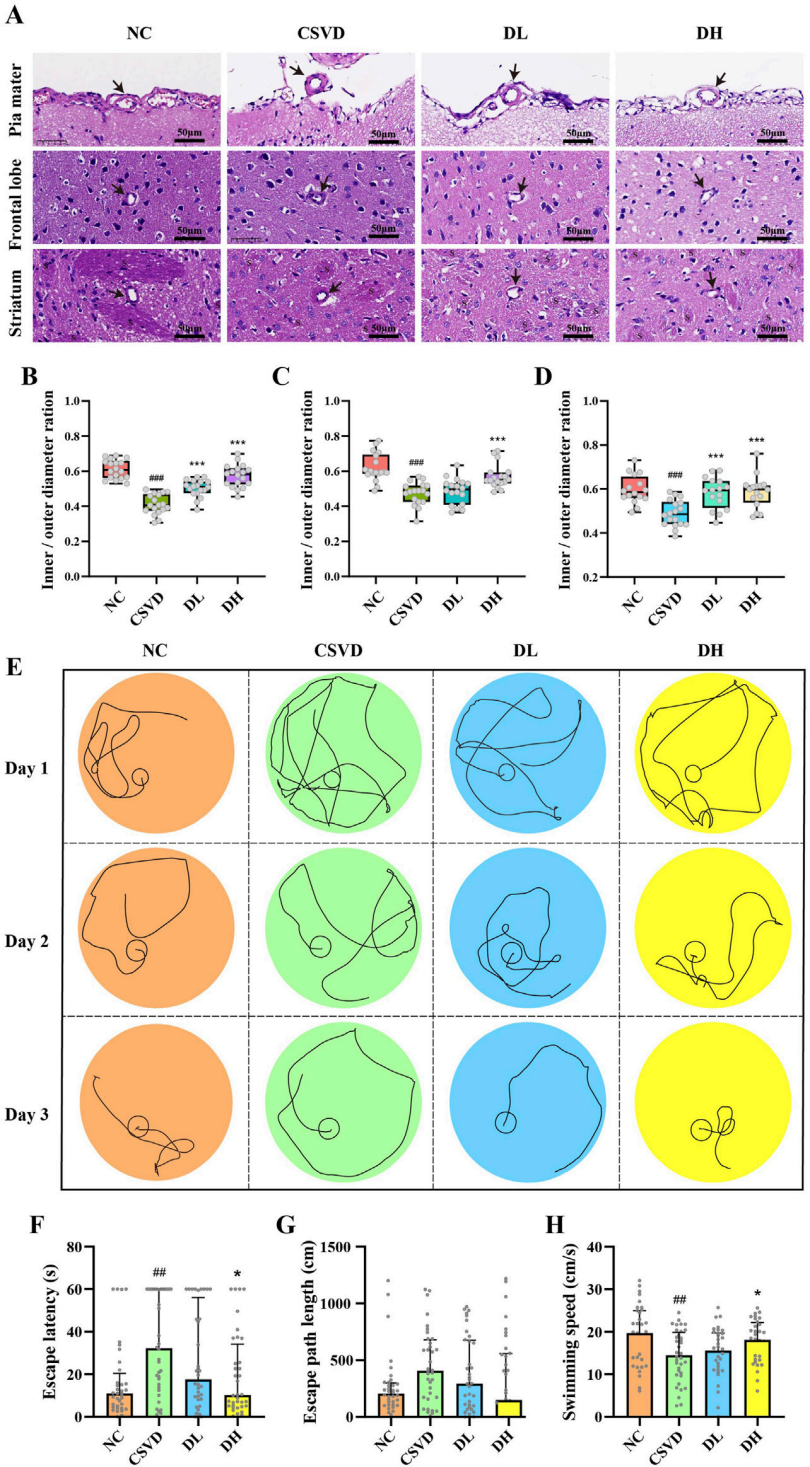
DHBF reduces the inner/outer diameter ratio of cerebral small vessels and improves the learning and memory ability of CSVD rats. **(A)** HE staining of cerebral small vessel in pia mater, frontal lobe, and striatum of CSVD rats. **(B–D)** Statistical analysis of inner/outer diameter ratio of pia mater **(B)**, frontal lobe **(C)**, and striatum **(D)**, n = 16–18. **(E)** Morris Water Maze Test evaluated the learning and memory ability of CSVD rats. **(F–H)** Statistical analysis of escape latency **(F)**, escape path length **(G)**, and swimming speed **(H)**, n = 36. Data were expressed by mean ± SD, compared with NC group, ##p < 0.01, ###p < 0.001; Compared with CSVD group, *p < 0.05, ***p < 0.001.

Previous studies have proved the possibility of 28-week-old SHR rats as a model for CSVD (Fu et al., 2023). As shown in Figures 1A–D, the vessel walls of small arteries in the pia mater, frontal lobe, and striatum of the CSVD group exhibited significant thickening (*p* < 0.001), with the ratios between the inner and outer diameter of these vessels were greatly decreased. This results suggested the successful establishment of the CSVD rat model. Notably, we observed that DHBF, especially at the high dose, significantly reversed this reduction (*p* < 0.001). These results suggested that DHBF could alleviate arteriolosclerosis in CSVD rats.

As shown in Figure 1E, the spatial acquisition trial showed a progressive reduction in the movement trajectories of all rats over time. As expected, these changes were more obvious in the NC and DH groups. On day 1, the CSVD, DL, and DH groups primarily used the random search strategy, characterized by an unbiased exploration of the entire area, while the NC group exhibited a focal search strategy (searching within a limited space). On day 2, the NC group showed a clearer search path, which was defined as an indirect search that primarily involved misdirected target searches. In contrast, the DL and DH groups translated into a scanning search strategy, which entailed random searching and avoiding walls. On day 3, both the NC and DH groups transitioned to a direct search strategy, characterized by movement trajectories aimed directly to the target. The CSVD and DL groups showed a chaining search strategy, which refers to a search along the pool wall. Additionally, the escape latency of the CSVD group showed a significant increase (Figure 1F, *p* < 0.01), while it was significantly reduced by about 68.4% in the DH group (*p* < 0.01). Although DHBF did not affect the escape path length (Figure 1G), the swimming speed of the DH group significantly recovered to the NC group level (Figure 1H, *p* < 0.01). These results indicated that the learning and memory abilities of CSVD rats were significantly decreased, while DHBF significantly enhanced these cognitive functions.

### 3.2 DHBF relieves prefrontal cortex and hippocampus injury in CSVD rats

**FIGURE 2:**
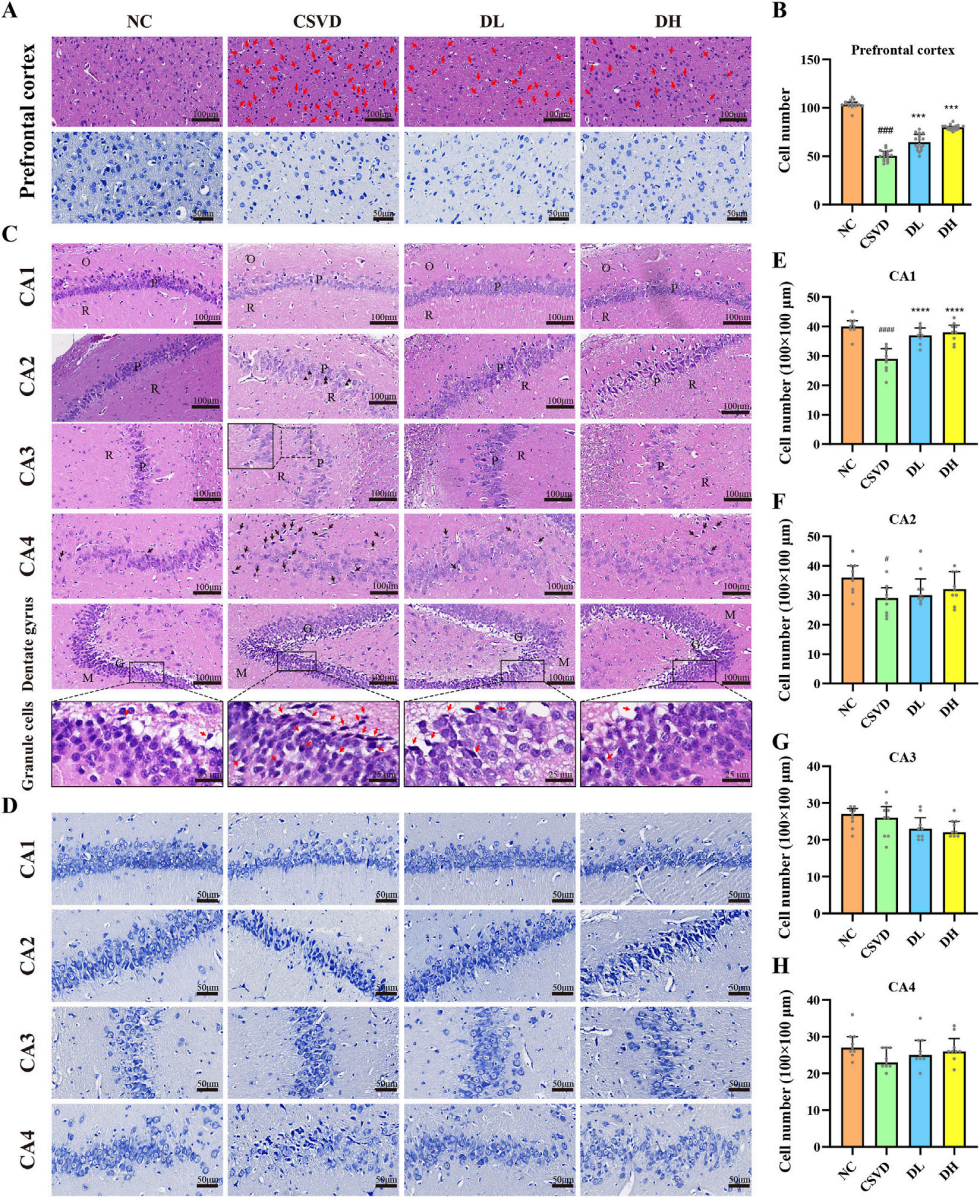
DHBF relieves prefrontal cortex and hippocampus injury in CSVD rats. **(A)** HE and Nissl staining of the prefrontal cortex. Karyopyknosis is shown by the red arrow. **(B)** Statistical analysis of cell number in the prefrontal cortex. **(C, D)** HE and Nissl staining of the hippocampus, including CA1 to CA4 and dentate gyrus. The swollen stratum radiatum fibers are shown by the black triangle and dotted box in CA2 and CA3, respectively. Karyopyknosis in CA4 is shown by the black arrow. The red arrow indicates karyopyknosis of the dentate gyrus granules of the hippocampus. O: polymorphic layer; P: pyramidal layer; R: radiatum layer; M: molecular layer; G: granular cell. **(E–H)** Statistical analysis of cell number in CA1 **(E)**, CA2 **(F)**, CA3 **(G)**, and CA4 **(H)**. Data were expressed by mean ± SD, compared with NC group, ^#^ *p* < 0.05, ^###^ *p* < 0.001, ^####^ *p* < 0.0001; Compared with CSVD group, ^***^ *p* < 0.001, ^****^ *p* < 0.0001, n = 9.

To investigate the influence of DHBF on the prefrontal cortex and hippocampus of CSVD rats, HE staining was used to observe the morphology of these brain regions, while Nissl staining was employed to evaluate the number of nerve cells. The prefrontal cortex of the CSVD group showed nuclear condensation, loss of nucleolus, and a decrease in the number of cells compared to the NC group. These changes were significantly alleviated after DHBF treatment (Figure 2A). Nissl staining indicated that the CSVD group had significantly fewer prefrontal neurons than the NC group (*p* < 0.001), while the DH group showed a significant increase in neuron count (Figure 2B, *p* < 0.001).

In addition, HE staining of the hippocampus showed a reduction in cell count and loosely arranged nuclei in the CSVD group, while the DL and DH group had varying degrees of improvement (Figure 2C). Moreover, the stratum radiatum fibers in the CA2 and CA3 regions of the CSVD group were swollen, which were significantly reduced after DHBF intervention (black triangle and dotted box). Furthermore, there was noticeable nuclear condensation and necrosis in the CA4 region of the CSVD group, which were significantly reduced in the DL and DH groups (black arrow). The overall structure of the dentate gyrus in each group was complete under the field of view of ×200 magnification. At 800 × magnification, a small amount of nuclear condensation was observed in the NC group, while there was a significant increase in the CSVD group (red arrow). Following DHBF intervention, nuclear condensation was significantly ameliorated. Compared to the NC group, the number of neurons in the CA1, CA2, and CA4 regions of the hippocampus in the CSVD group were significantly decreased, especially in the CA1 (Figures 2D–H, *p* < 0.001). DHBF caused a significant increase in the number of neurons within the CA1 region of the hippocampus (*p* < 0.001). In summary, DHBF could relieve prefrontal cortex and hippocampus injury and increase the number of nerve cells in CSVD rats.

### 3.3 DHBF increased cerebral blood perfusion and oxygen supply capacity in CSVD rats

**FIGURE 3:**
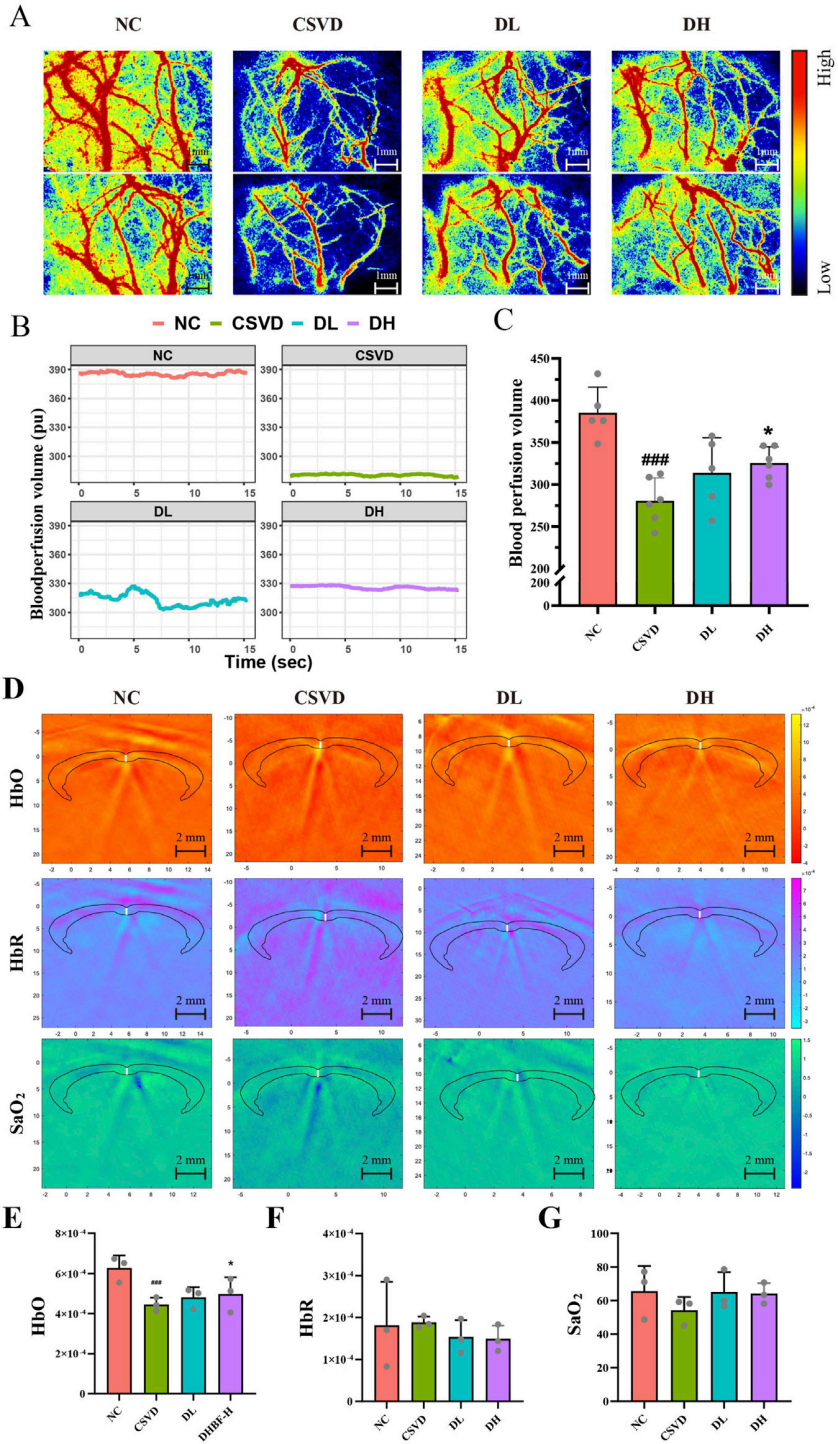
Cerebral blood perfusion and oxygen supply capacity with LSCI and PAT in CSVD rats. **(A)** Representative images of CBF by LSCI in different groups. **(B, C)** Quantitative analysis of CBF for 15s, n = 5–6. **(D)** PAT reconstruction images of the brain. Black and white lines indicate the skull and the sagittal suture, respectively. **(E–G)** Statistical analysis of HbO **(E)**, HbR **(F)**, and SaO_2_ **(G)**, n = 3. Data were expressed by mean ± SD, compared with the NC group, ^###^ *p* < 0.001; Compared with the CSVD group, ^*^ *p* < 0.05.

To determine the effects of DHBF on cerebral blood perfusion volume and cortical oxygen supply capacity in CSVD rats, we used LSCI and PAT to detect the changes in cerebral blood flow. As shown in Figure 3A, the NC group showed abundant cerebral vessels with good blood perfusion, while the CSVD group displayed sparse vessels, thinner tube diameter, significantly decreased perfusion, and obvious areas of hypoperfusion. Although DHBF improved blood perfusion, significant areas of hypoperfusion remained in the DL group. The cerebral perfusion volume was quantitatively measured at 15 s (Figures 3B,C). The study found that the average cerebral blood flow perfusion volume was 385.2 pu in the NC group, which significantly decreased to 280.4 pu in the CSVD group (*p* < 0.001). After DHBF intervention, the DL group increased to 313.6 pu, while the DH group dramatically improved to 325.5 pu (*p* < 0.05). In addition, PAT results showed a significant reduction of the oxyhemoglobin (HbO) levels in the CSVD group (Figure 3D, *p* < 0.001), and a substantial increase to 3.5 × 10^−4^ in the DH group (*p* < 0.05). Although there was no statistical difference in cerebral cortex oxygen saturation (SaO_2_) among all groups, the trend was consistent with that of HbO. These results showed that DHBF could increase cerebral blood perfusion and oxygen supply capacity in CSVD rats.

### 3.4 Enrichment analysis and PPI network construction

To reveal the functions and enrichment pathways of anti-CSVD targets of DHBF, GO, and KEGG enrichment analyses were performed. The UPLC-Q-Orbitrap HRMS was adopted to identify the composition of DHBF. All data were obtained under positive and negative modes via the full MS/dd-MS2 channel and analyzed using the mzClound and the mzVault network database. The compounds obtained from the UPLC were screened at SwissADME (http://www.swissadme.ch/) for oral bioavailability, druglikeness and blood-brain barrier, and the final screening yielded five compound compositions, which are shown in Supplementary Table S3, and the qualified compounds were used for web-based pharmacological analysis.

**FIGURE 4:**
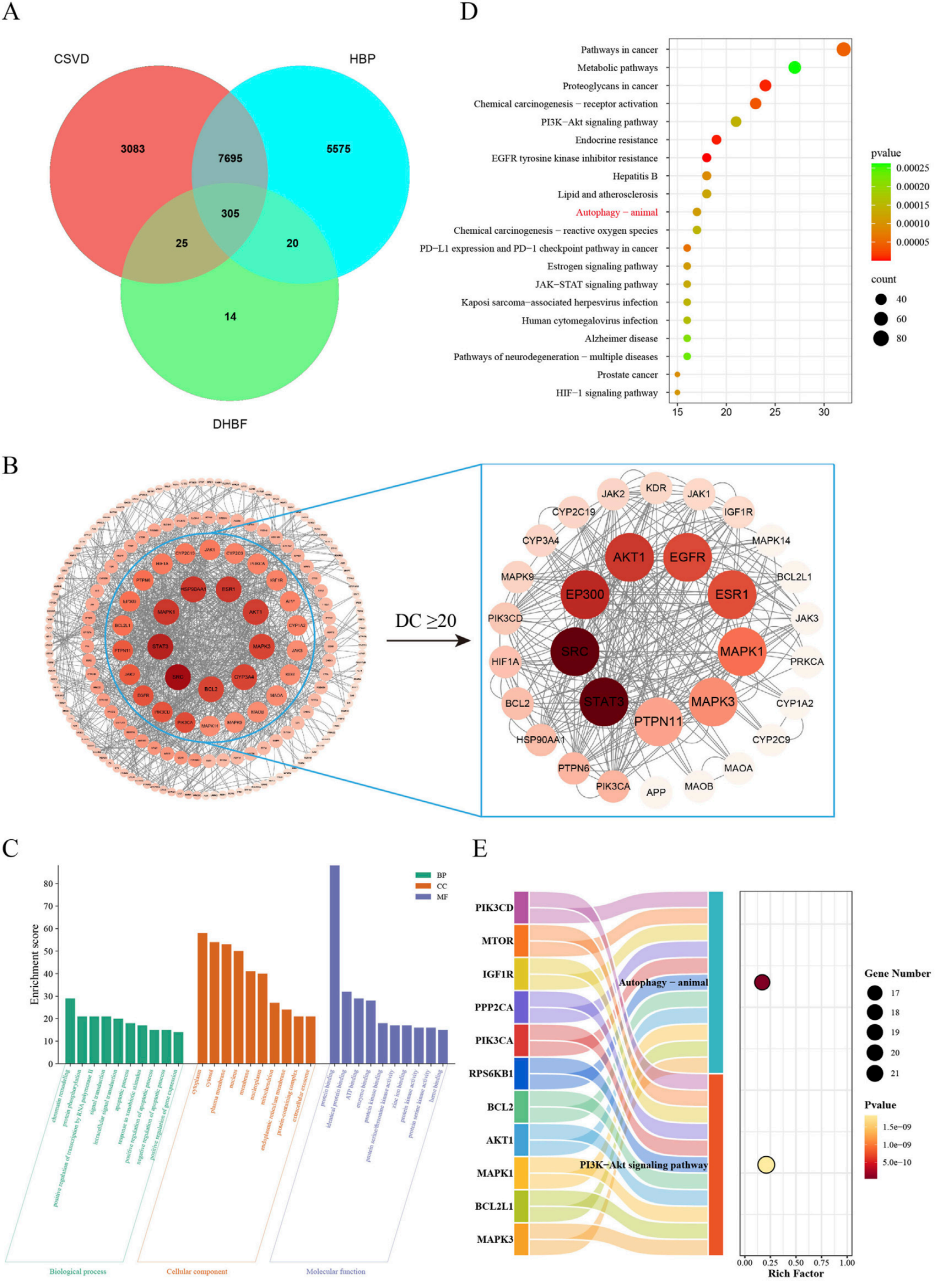
Network analysis of DHBF treatment for CSVD. **(A)** Intersection targets of CSVD, HBP, and DHBF. **(B)** topological analysis of PPI interaction network. 305 protein nodes were obtained according to the intersection targets, and after screening by DC ≥20, a total of 31 protein nodes were obtained. **(C)** The bar diagram of GO enrichment analysis of DHBF-CSVD-HBP genes, including the top 10 GO terms of BP, CC, and MF. **(D)** The bubble diagram of the top 20 terms of KEGG enrichment pathways. The larger size of a dot indicates the larger number of genes annotated in the pathway, and the redder color of a dot stands for the lower q value. **(E)** The Sankey diagram reveals the relationship between targets and pathways.

A total of 364 target genes of active ingredients were obtained from SwissTargetPrediction. From GeneCards, 13595 and 11108 related genes were retrieved with “hypertension” and “cerebral small vessel disease” as the keywords, respectively. Consequently, 8,000 targets were obtained as the disease targets of hypertension combined with cerebral small vessel disease. There were 305 intersection targets among drug and disease targets that were considered central to subsequent studies (Figure 4A). The PPI network which contains 305 nodes and 558 edges was visualized and analyzed by Cytoscape 3.9.1 (Figure 4B). A total of 305 nodes were sorted by DC value, which was proportional to the size and color of the nodes. The key nodes for DHBF against CSVD were screened with DC ≥ 20 and sorted according to BC value which contains 31 nodes and 258 edges. The nodes with the highest BC values were STAT3, SRC, EP300, AKT1, EGFR, ESR1, MAPK1, MAPK3, and PTPN11, which may be crucial in the treatment of CSVD of DHBF.

There were 810 statistically GO terms, including 544 of biological processes (BP), 87 of cellular components (CC), and 179 of molecular function (MF). The top 10 important GO terms of BP, CC, and MF with the highest gene counts were visualized in Figure 4C. The result showed that BP was mainly related to chromatin remodeling, protein phosphorylation, and positive regulation of transcription by RNA polymerase II. CC was primarily associated with the cytoplasm, cytosol, and plasma membrane. MF was implicated in protein binding, identical protein binding, and ATP binding. Additionally, KEGG analysis showed 160 statistically significant pathways, and the top 20 significant pathways were displayed in Figure 4D. These results suggested that the mechanism by which DHBF treats CSVD is concentrated in PI3K-Akt signaling pathway, autophagy–animal, estrogen signaling pathway, JAK-STAT signaling pathway and HIF-1 signaling pathway. It has been shown that Angs and Tie2, as epidermal growth factor and epidermal growth factor receptor, are involved in the regulation of the PI3K-Akt signaling pathway (Chen et al., 2023). And recent study have indicated that autophagy levels were found to be significantly elevated following antagonism of Tie2, while agonism of ANG1 inhibited autophagy (Wu et al., 2024). Additionally, PI3K-Akt signaling pathway (Liang et al., 2023), JAK-STAT signaling pathway (You et al., 2015; Chen et al., 2021) and HIF-1 signaling pathway (Lu et al., 2018) are closely associated with autophagy. In conclusion, with the evaluation metrics of Count value and *p* value taken into account, it is suggested that modulation of autophagy may be a direct manifestation of DHBF in the treatment of CSVD. Consequently, this study investigated the hypothesis that DHBF regulates autophagy through the Angs-Tie2 pathway.

### 3.5 DHBF promoted proliferation, migration, and invasion of HUVECs under hypoxia

**FIGURE 5:**
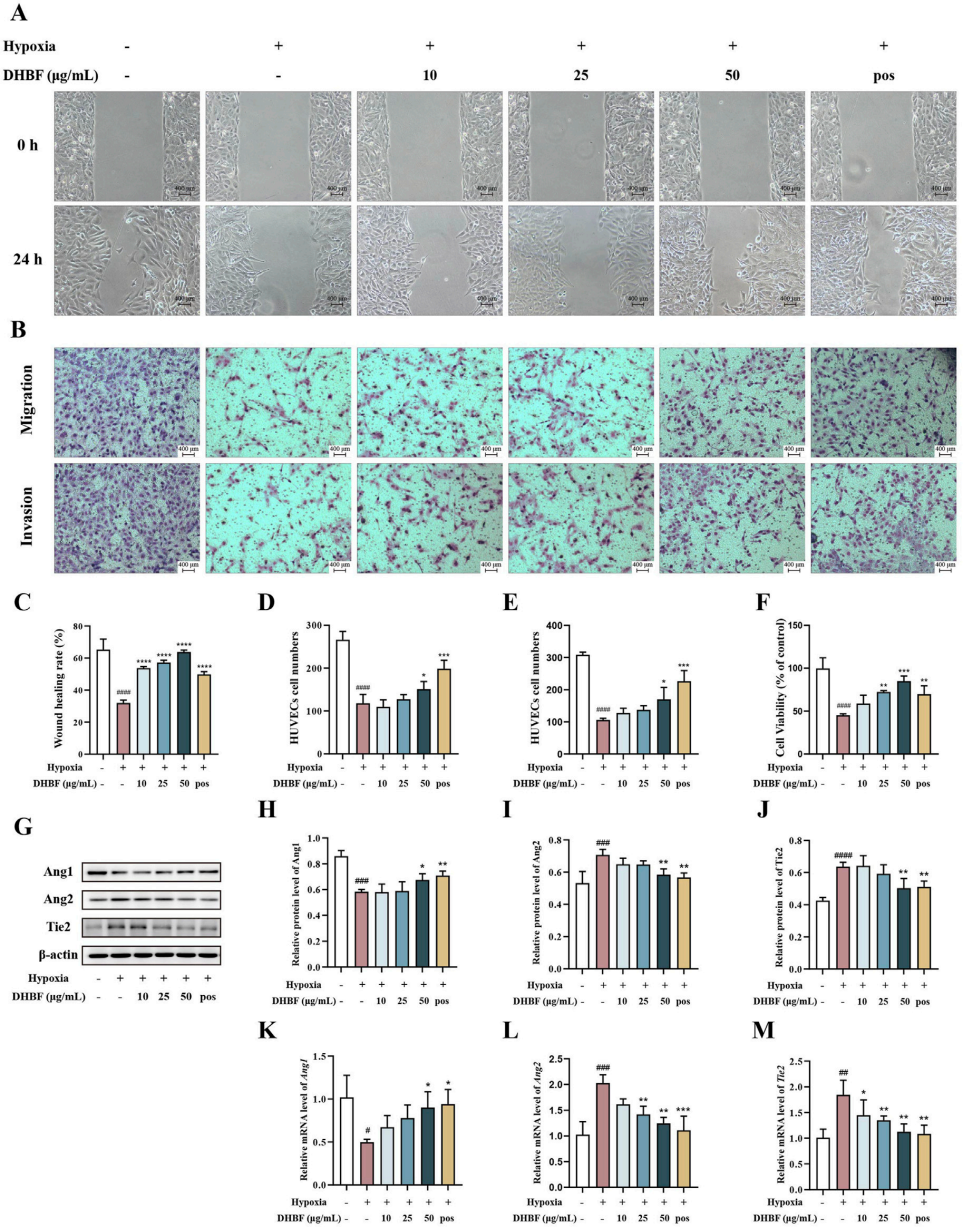
DHBF promoted proliferation, migration, and invasion and regulated the Angs-Tie2 pathway of hypoxia-induced HUVECs. **(A)** The wound healing ability of HUVECs was determined by the scratch assay with or without DHBF for 0 and 24 h. **(B)** Migration and invasion ability were measured by transwell assay. **(C)** Statistical analysis of the rate of wound healing. **(D)** Statistical analysis of migration ability. **(E)** Statistical analysis of invasion ability. **(F)** Effects of DHBF on the proliferation ability of HUVECs were determined by CCK-8 assay. **(G)** Western blot analysis of Ang1, Ang2, Tie2. Statistical analysis of the relative protein level of Ang1 **(H)**, Ang2 **(I)**, Tie2 **(J)**. **(K–M)** qRT-PCR analysis of *Ang1* **(K)**, *Ang2* **(L)**, *Tie2* **(M)**. Data were expressed by mean ± SD, compared with Control group, ^###^ *p* < 0.001; Compared with model group, ^*^ *p* < 0.05, ^**^ *p* < 0.01, ^***^ *p* < 0.001, ^****^ *p* < 0.0001, n = 3.

To investigate the effects of DHBF on the proliferation, migration, and invasion of HUVECs under hypoxic conditions, we performed wound healing, CCK-8, and transwell assay. The HUVECs were processed with different concentrations of DHBF and Nicorandil while exposed to a hypoxia environment. As illustrated by the representative images in Figure 5C, DHBF markedly promoted the proliferation of HUVECs under hypoxia (*p* < 0.001). Concomitantly, the cell viability of HUVECs was increased after 25 and 50 μg/mL DHBF treatment in the hypoxia environment (Figures 5F, *p* < 0.01). Additionally, DHBF dose-dependently accelerated the migration and invasion of HUVECs, as demonstrated by the transwell assay results (Figures 5D,E *p* < 0.05). These results suggested that DHBF markedly increased the viability, and promoted migration and invasion of HUVECs under hypoxia.

### 3.6 DHBF regulated Angs-Tie2 pathway and suppressed autophagy of HUVECs under hypoxia

The Angs-Tie2 pathway is recognized for its crucial role in regulating vascular stability, Ang1 acts as a Tie2 agonist that promotes vessel stability while Ang2 is a context-dependent weak Tie2 agonist or antagonist (Saharinen et al., 2017). As the Ang2 level is strongly correlated with vascular dysfunction diseases (Fagiani and Christofori, 2013), the effect of DHBF on Ang1, Ang2, and Tie2 expression was determined. As shown in Figure 5, the expression of Ang2 and Tie2 were notably restricted by DHBF compared with hypoxia HUVEC (*p* < 0.05). Additionally, DHBF showed the potential to increase the expression of Ang1 (*p* < 0.01). These findings indicate that DHBF increased the expression of Ang1 and restored the abnormal increase of Tie2 under hypoxia, which enhanced the stability of blood vessels.

**FIGURE 6:**
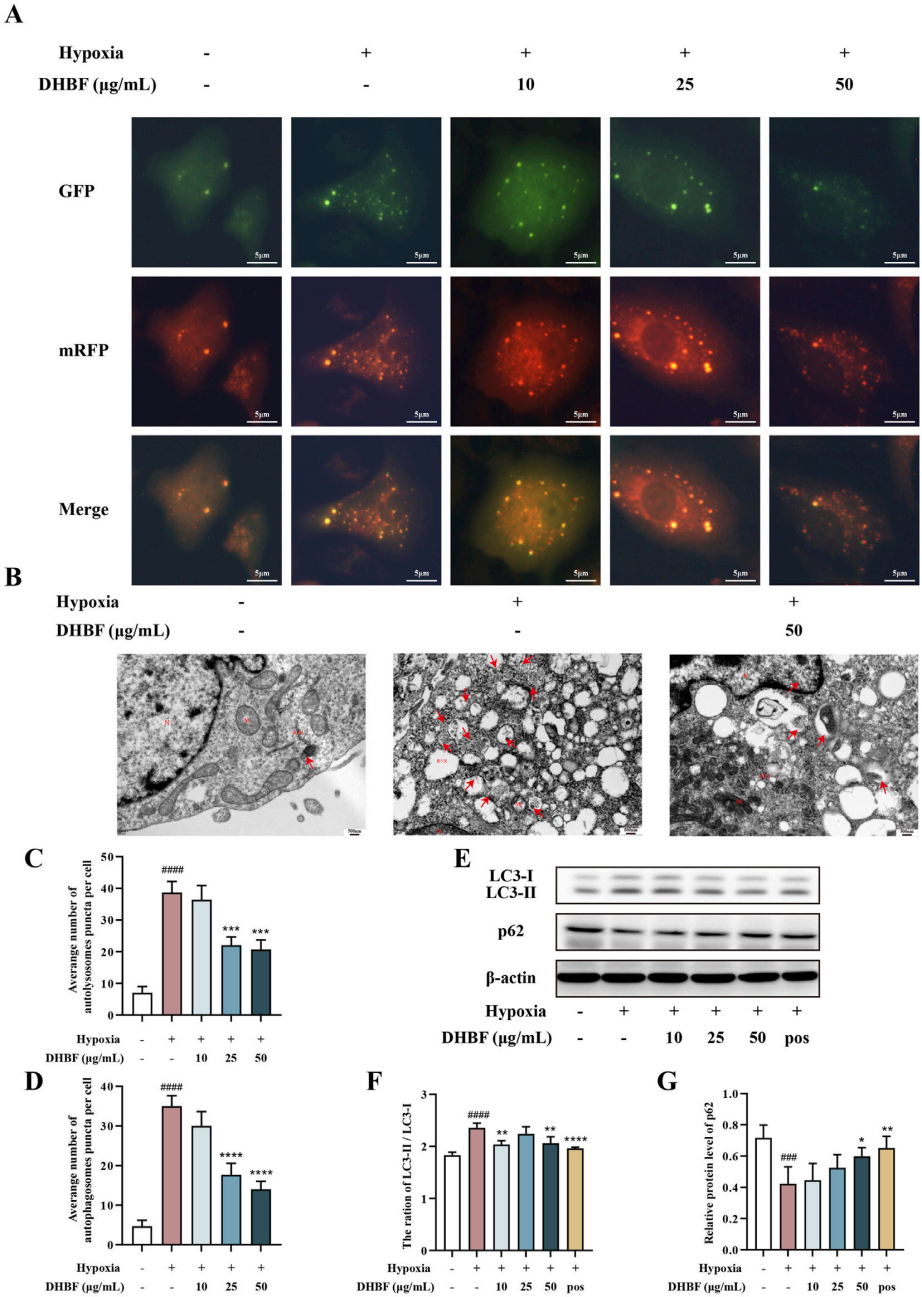
Effects of DHBF on autophagy in hypoxia HUVECs. **(A)** Laser scanning confocal microscopy for observing autophagy flux. Red puncta and yellow puncta represent autolysosomes and autophagosomes, respectively. **(B)** Transmission electron microscope for observing autolysosome (red arrow). **(C)** Statistical analysis of autolysosomes and **(D)** autophagosomes. **(E–G)** Western blot analysis of LC3-Ⅱ/LC3-Ⅰ and p62. Data was expressed by mean ± SD, compared with Control group, ^###^ *p* < 0.001, ^####^ *p* < 0.0001; Compared with model group, ^*^ *p* < 0.05, ^**^ *p* < 0.01, ^***^ *p* < 0.001, ^****^ *p* < 0.0001, n = 3.

Considering that excessive autophagy induces cell death and may promote the occurrence of CSVD, we assessed whether DHBF affected autophagy of HUVECs under hypoxia using the autophagy flux reporter mRFP-GFP tandem florescent-tagged LC3 adenovirus (Qin et al., 2022). Red fluorescence represented autolysosomes while yellow fluorescence represented autophagosomes (Figure 6A). As shown in Figures 6C,D, the red (mRFP^+^GFP^−^) and yellow (mRFP^+^GFP^+^) puncta per cell were greatly suppressed by DHBF in HUVECs under hypoxia (*p* < 0.001). In addition, the autophagy of HUVEC was monitored by TEM. There were only a few autolysosomes in the control group, whereas a large number of autolysosomes appeared under hypoxia regulation (Figure 6B). Importantly, the number of autophagy lysosomes decreased significantly after DHBF intervention, indicating that DHBF can inhibit autophagy under hypoxia. We next performed protein analysis on LC3 and p62, the important biomarkers of autophagy (Cao et al., 2019). As shown in Figures 6E–G, DHBF not only decreased the LC3-Ⅱ/LC3-Ⅰ ratio but also upregulated the p62 (*P* < 0.05). In summary, these results suggested that DHBF suppressed autophagy with increased expression of p62 and decreased the LC3-Ⅱ/LC3-Ⅰ ratio.

### 3.7 Ang2 restricted proliferation, migration, and invasion of HUVECs under hypoxia

**FIGURE 7:**
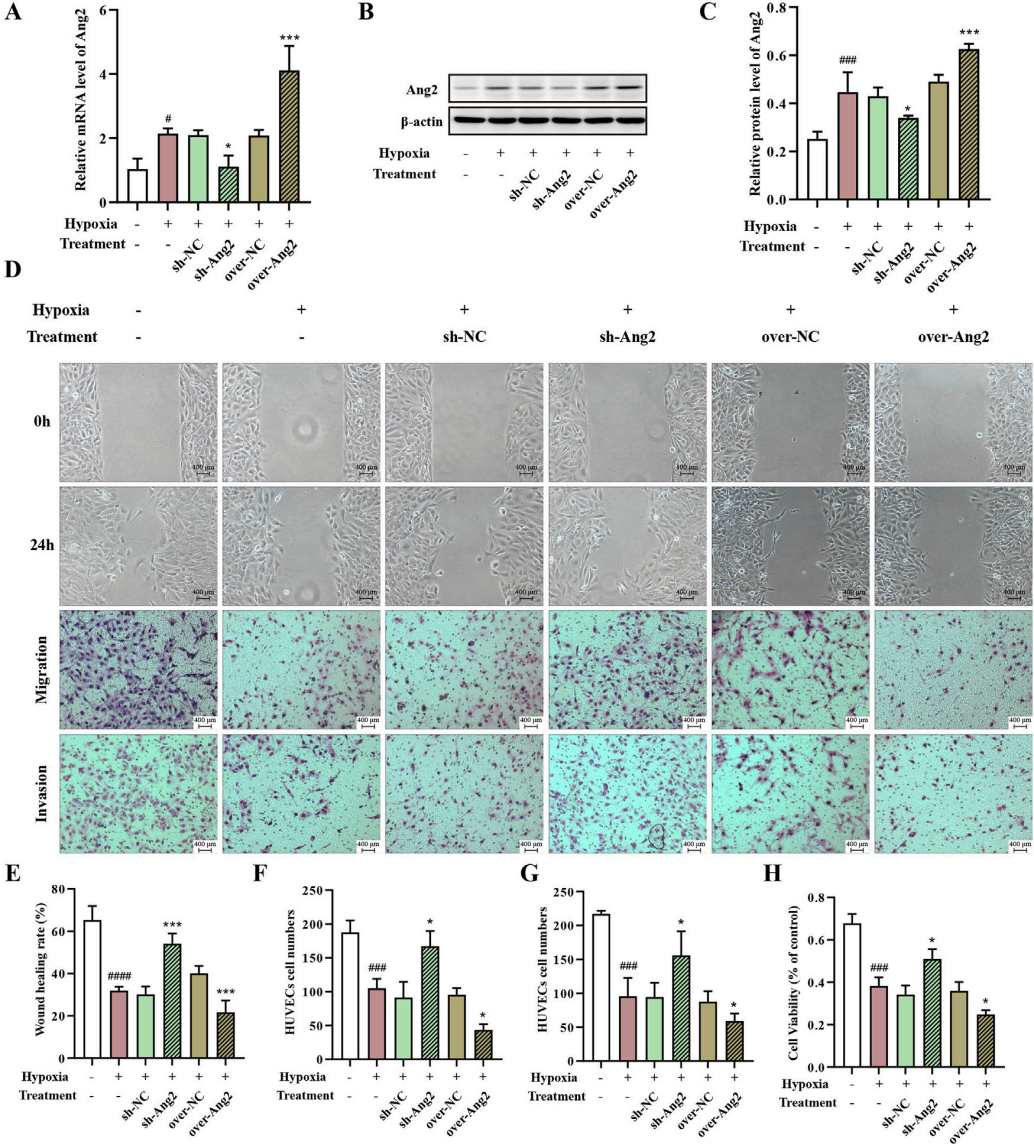
The role of Ang2 on proliferation, migration, and invasion ability of hypoxia HUVECs. **(A–C)** Overexpression and knockdown of Ang2 were verified by Western blot **(A–B)** and qRT-PCR **(C)**. **(D)** Scratch assay and transwell assay of HUVECs with overexpression and knockdown of Ang2. Statistical analysis of wound healing **(E)**, migration ability **(F)**, and invasion ability **(G)**. **(H)** The proliferation ability of HUVECs with overexpression and knockdown of Ang2 was determined by CCK-8 assay. Data were expressed by mean ± SD, compared with Control group, ^#^ *p* < 0.05, ^###^ *p* < 0.001, ^####^ *p* < 0.0001; Compared with model group, ^*^ *p* < 0.05, ^***^ *p* < 0.001, n = 3.

We have found that DHBF enhanced the viability, migration, and invasion of HUVECs under hypoxia, and notably decreased the expression of Ang2, but the role of Ang2 is still unclear. We therefore knocked down and overexpressed Ang2 by lentiviral transduction, respectively. As shown in Figures 7A–C, the successful knockdown and overexpression of Ang2 were verified by Western blot and qRT-PCR (*p* < 0.05). Notably, the inhibition of Ang2 expression accelerated the cell migration, invasion, and proliferation under hypoxia (Figures 7D–G, *p* < 0.05). While overexpression of Ang2 could reverse this trend (*p* < 0.05). To summarize, these results revealed that Ang2 restricted the proliferation, migration, and invasion of HUVECs under hypoxia.

### 3.8 Ang2 induced autophagy in HUVECs under hypoxia

**FIGURE 8:**
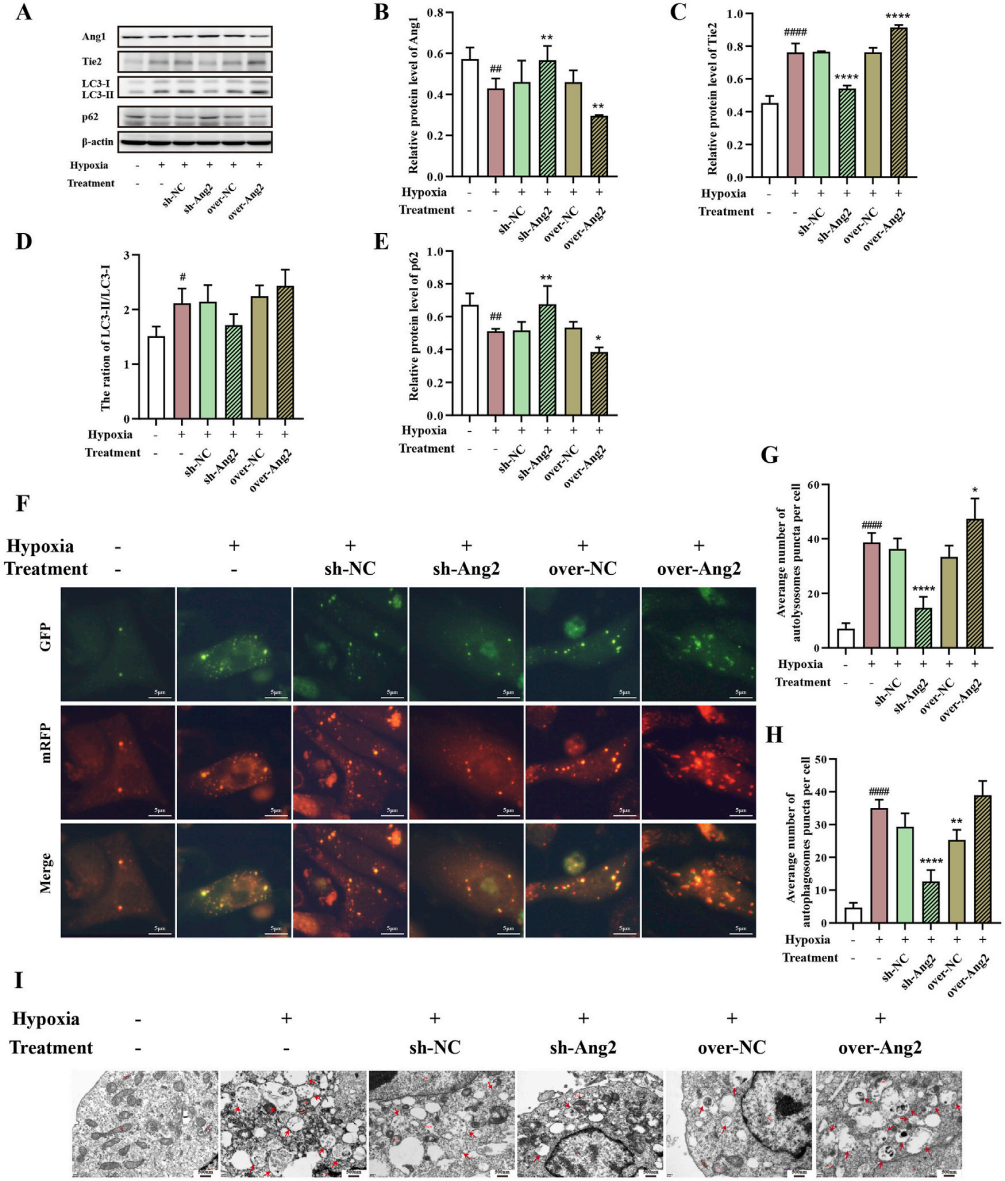
The role of Ang2 on the Angs-Tie2 pathway and autophagy of hypoxia HUVECs. **(A–F)** Western blot analysis of Ang1 **(B)**, Tie2 **(C)**, LC3-Ⅱ/LC3-Ⅰ **(D)**, and p62 **(E)**. **(F–H)** autophagy flux of Ang2 overexpression and knockdown HUVEC was observed by laser scanning confocal microscopy. Red puncta and yellow puncta represent autolysosomes and autophagosomes, respectively. Statistical analysis of autolysosomes **(G)** and autophagosomes **(H)**. **(I)** Transmission electron microscope for observing autolysosome (red arrow). Data was expressed by mean ± SD, compared with Control group, ^###^ *p* < 0.001, ^####^ *p* < 0.0001; Compared with model group, ^*^ *p* < 0.05, ^**^ *p* < 0.01, ^***^ *p* < 0.001, ^****^ *p* < 0.0001, n = 3.

To identify the role of Ang2 in regulating autophagy, we analyzed the impacts of Ang2 knocking down or overexpressing on the autophagy of HUVECs under hypoxic conditions. We found that knockdown of Ang2 in hypoxia conditions resulted in the reduction of Tie2 expression and increased Ang1 expression, whereas the opposite result was observed after overexpression of Ang2 (Figures 8A‐C *, p* < 0.05). Furthermore, the LC3-Ⅱ/LC3-Ⅰ ratio was downregulated and the expression of p62 was elevated after the knockdown of Ang2, while overexpression of Ang2 presented the opposite trend (Figures 8D,E, *p* < 0.05). To further monitor autophagic flux, dual fluorescence analysis with mRFP-GFP-LC3 was adopted. As shown in Figures 8F–H, the knockdown of Ang2 decreased the accumulation of red and yellow dots while significantly increased after overexpression of Ang2. In addition, TEM showed that autophagy increased under hypoxia after Ang2 overexpression, while autophagy decreased under hypoxia after Ang2 knockdown (Figure 8I). Therefore, these results indicated that Ang2 promoted autophagy in HUVECs under hypoxia.

### 3.9 DHBF inhibited autophagy in hypoxia HUVECs via downregulation of Ang2

**FIGURE 9:**
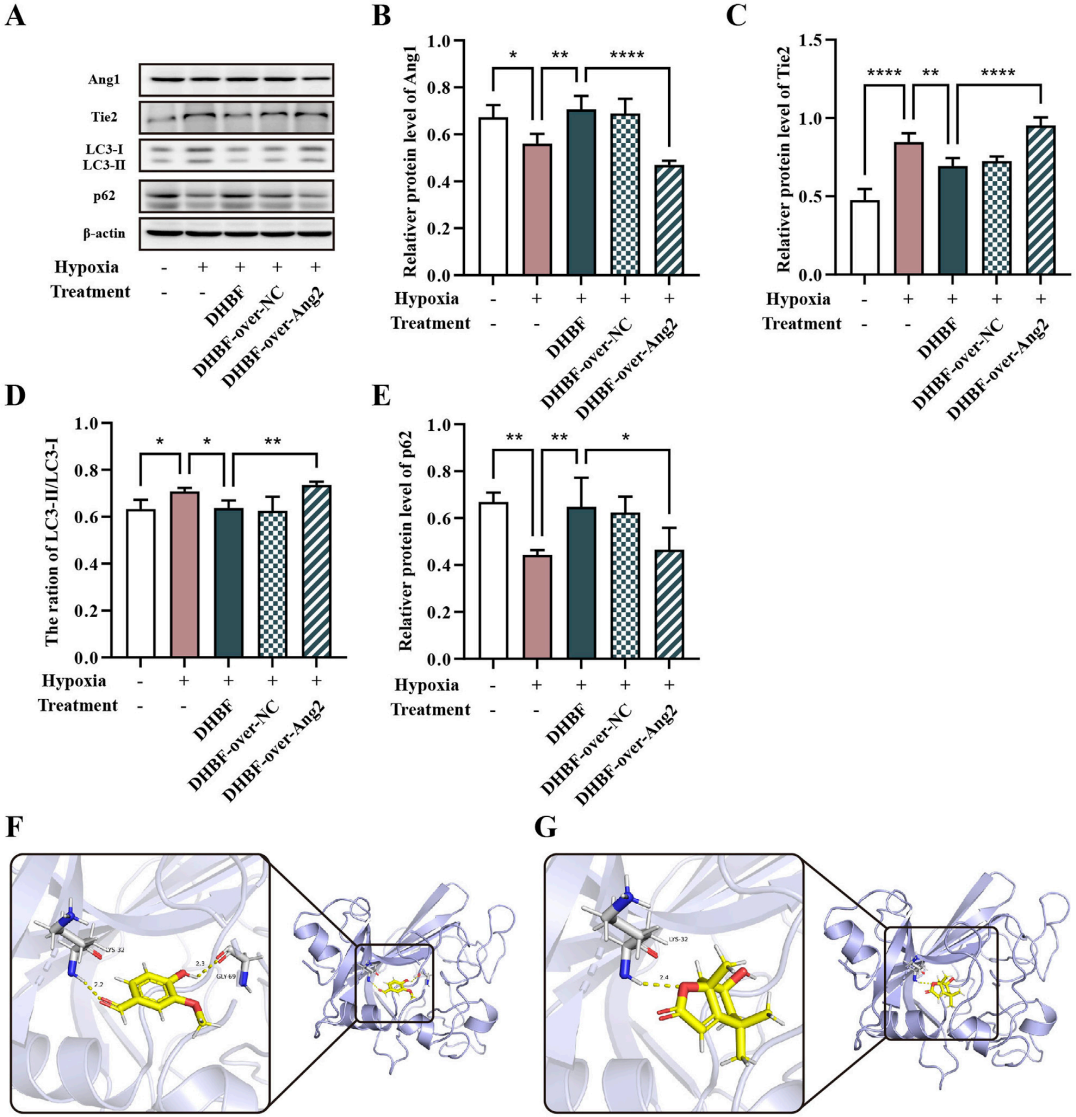
DHBF inhibited autophagy and regulated the Ang1-Tie2 pathway in hypoxia HUVECs via targeting downregulation of Ang2 and molecular docking models of Ang2 to DHBF. **(A–F)** Western blot analysis of Ang1 **(B)**, Tie2 **(C)**, LC3-Ⅱ/LC3-Ⅰ **(D)**, and p62 **(E)**. Molecular docking models of Ang2 to 2 ingredients of DHBF. Vanilin **(F)** and Loliolide **(G)**. Data was expressed by mean ± SD, ^*^ *p* < 0.05, ^**^ *p* < 0.01, ^****^ *p* < 0.0001, n = 3.

To further confirm the role of Ang2 in DHBF affecting the HUVECs under hypoxia, we overexpressed Ang2 before treatment with a high dose of DHBF under hypoxia conditions. Overexpression of Ang2 followed by DHBF treatment significantly restricted the expression of Ang1 compared to the DHBF group (Figures 9B, *p* < 0.001). Additionally, the regulations of the LC3-Ⅱ/LC3-Ⅰ ratio and p62 by DHBF were also inhibited after Ang2 overexpression (Figures 9D,E, *p* < 0.01). Overall, these results proved that DHBF inhibited autophagy in HUVECs under hypoxia via downregulation of Ang2.

### 3.10 Molecular docking screening of active ingredients for DHBF targeted Ang2

To preliminarily verify the targeting effect of DHBF on Ang2 and to screen the effective components for subsequent experiments, we performed molecular docking of Ang2 protein with the components identified from DHBF. Ang2 was found to interact with loliolide and vanillin, and the binding energy values were smaller than −5 kJ/mol, which was considered to have good binding activity (Figure 9). These results suggested that DHBF might inhibit Ang2 to maintain the stability of vascular and restrictions on autophagy promote cell survival by inhibiting Ang2.

## 4 Discussion

The prevalence of CSVD increases with age, ranging from 5% in individuals aged 50 to nearly 100% in those over 90 (Cannistraro et al., 2019). Current treatments for CSVD primarily focus on stroke and cognitive impairment, while specific treatments for CSVD patients are still lacking. In our study, SHR rats were used to explore the effect of DHBF on VCI and small vascular lesions in CSVD, since SHR rats have been validated as a disease model for CSVD (Fu et al., 2023). In this study, we proved that DHBF could relieve VCI and vascular lesions in CSVD rats, possibly by targeting Ang2 to regulate the Angs-Tie2 pathway and endothelial autophagy. These results provide new medicine and potential therapeutic targets for the treatment of CSVD and provide new insights for subsequent drug development of CSVD.

The prefrontal cortex and hippocampus are widely recognized as critical to cognitive function (Jenkins, 2022), with the hippocampus being critical for spatial and episodic memory (Lisman et al., 2017), and the prefrontal cortex being important for receiving signals and making connections between thinking and acting (Miller and Cohen, 2001). Our results suggest that CSVD was impaired in learning and memory, and DHBF significantly improved VCI by alleviating hippocampal and prefrontal lobe damage in CSVD. In addition, CSVD caused by arteriolosclerosis is characterized by vascular wall thickening and lumen stenosis, which is associated with abnormal cerebral blood perfusion (Pantoni, 2010). Our results showed that CSVD presents low perfusion areas and significantly reduced oxygen supply capacity, while DHBF treatment enhanced the blood perfusion and oxygen supply capacity. Moreover, the results of cerebral arteriole vascular staining showed that DHBF has a protective effect against vascular lesions associated with CSVD. It is worth noting that this is the first report on DHBF treatment of CSVD.

To explore the effective ingredients of DHBF in the therapeutic of CSVD, the components of DHBF were identified by UPLC-Q-Orbitrap HRMS and screened on the SwissADME. Among them, erucamide (Mitchell et al., 1996) and vanillin (Elseweidy et al., 2023) have been reported to promote angiogenesis. Furthermore, carvone has been reported to be cardioprotective through antioxidative stress (Abbas et al., 2020). Caryophyllene has been demonstrated to protect the neurovascular against the hypoxia-reperfusion injury *in vitro* (Tian et al., 2016). In conclusion, these components may be the effective ingredients of DHBF.

To explore the potential mechanism of DHBF against CSVD, a network pharmacological analysis of DHBF and CSVD was conducted. The KEGG results revealed that their intersection targets were mainly enriched in the PI3K-Akt pathway and autophagy–animal (Figure 4D). Studies have shown that vascular endothelial dysfunction precedes other pathologies in CSVD (Rajani et al., 2018) and is a main determinant of structural and functional changes in the cerebral vascular (Yang et al., 2022). Consistently high levels of autophagy may play an important role in vascular endothelial dysfunction, and the inhibition of autophagy has been shown to attenuate endothelial dysfunction in HUVEC (Xu et al., 2024). Furthermore, the activation of the PI3K-Akt pathway has been shown to inhibit autophagy and significantly reduce brain damage caused by chronic cerebral hypoperfusion (Hu et al., 2017). Additionally, the PI3K-Akt signaling pathway is closely associated with the Angs-Tie2 pathway. Studies have demonstrated that ANG1 can promote endothelial cell survival through the activation of the PI3K pathway, with PI3′-kinase and AKT being crucial elements of the Ang1 pathway (Kim et al., 2000). Besides, Ang1 activates AKT phosphorylation in HUVECs in a PI3K-dependent manner (Babaei et al., 2003). Accordingly, inhibit excessive autophagy may represent a potential therapeutic mechanism for the treatment of CSVD by DHBF. However, further research is required to verify whether DHBF inhibits autophagy through the activation of the PI3K-Akt pathway.

Our results in this study demonstrate the successful simulation of the cerebral microvascular environment in CSVD patients, effectively establishing an *in vitro* cell model of mild hypoxic injury of vascular endothelium. HUVEC cells have been widely used to explore the mechanism of endothelial dysfunction-related diseases. However, the common use of intermittent hypoxia, 1% O_2_, or even completely anaerobic culture conditions does not accurately reflect the mild hypoxia of CSVD patients. Therefore, our research used a 10% O_2_ intervention in HUVECs, which better mimics the cerebral ischemia in patients with CSVD. As shown in Figure 5, the proliferation, migration, and invasion abilities of HUVECs in the model group were significantly impaired. This may be related to the increased Ang1 and decreased Ang2 following DHBF treatment. Ang1, a potent angiogenic factor, activates the phosphorylation of Tie2 with a function distinct from VEGF (Akwii et al., 2019). Ang2 competitively targets Tie2 and suppresses Ang1-induced phosphorylation of Tie2 (Maisonpierre et al., 1997), but Ang2 also induces phosphorylation of Tie2 under certain circumstances, albeit with a weaker intensity than Ang1 (Yuan et al., 2009). Additionally, Ang2 has been shown to play a role in the modulation of angiogenic sprouting and vessel regression (Gale et al., 2002). Elevated level of Ang2 in plasma was associated with endothelial dysfunction, the decrease of Ang2 level reflects the improvement of vascular state after treatment (Saharinen et al., 2017). This was in line with our results.

On the other hand, recent studies have shown that Ang1 protects nerves by inhibiting autophagy (Yin et al., 2019). Nevertheless, autophagy is a double-edged sword that maintains cell homeostasis and functions by recycling needless and dysfunctional substances or organelles. The absence of autophagy generally induces small blood vessel impairment (Nguyen et al., 2018). However, excessive autophagy induced by hypoxia and stress can also lead to cell death, which is known as type II programmed death (Bravo-San Pedro et al., 2017). Excessive autophagy has been demonstrated to play a pivotal role in the inflammatory damage to HUVECs (Chen et al., 2024). Notably, excessive autophagy has been reported to be an important factor in pulmonary vascular remodeling, leading to thickening of the vessel wall (Zhang et al., 2023). In addition, the inhibition of excessive autophagy has been widely discussed in the treatment of vascular diseases. For instance, Lauric acid-induced CSVD has been demonstrated to be associated with excessive autophagy while inhibition of autophagy has been demonstrated to ameliorate CSVD (Zhang et al., 2017). Additionally, inhibition of autophagy effectively alleviates dysfunction of mesenteric arteries in hypertension, reducing blood pressure and vessel wall thickness, and exerting a direct vasodilatory effect (Kwon et al., 2022). Furthermore, inhibition of autophagy has been reported to be a potent therapeutic strategy for mitigating ischemic brain injury during the ischemia/reperfusion (Dong et al., 2017). Consequently, the inhibition of autophagy is hypothesized to be a therapeutic strategy for CSVD that may act through multiple mechanisms, including the potential to lower blood pressure, reduce inflammation, impede vessel wall remodeling, diastole vessel, and attenuate brain damage.

In the present study, we found that DHBF could reduce hypoxia-induced autophagy in HUVECs, indicating that DHBF may alleviate cell damage by inhibiting endothelial autophagy, thereby enhancing cell viability. In addition, we noted that DHBF decreased Ang2 expression. Ang2 has been shown to promote autophagy in a dose-dependent manner in previous studies (Yin et al., 2018). Based on this above evidence, we knocked down and overexpressed Ang2 to observe the effects of Ang2 on DHBF regulation of autophagy. The results suggested that Ang2 participated in the regulation of autophagy, and DHBF inhibited autophagy and angiogenesis in HUVECs by down-regulating Ang2. Molecular docking analysis suggested that this effect may be closely associated with loliolide and vanillin.

There are several limitations in this study. First, we did not validate the mechanism by which DHBF ameliorates CSVD in animal models. Further studies are necessary to clarify the mechanism of DHBF action *in vivo* by detecting the expression of Ang2 and related indicators in CSVD rats. Second, we did not knock down or overexpress Ang2 in CSVD rats to verify whether DHBF works by targeting Ang2 *in vivo*. Third, we hypothesize that autophagy is one of the potential mechanisms for the treatment of CSVD. However, we did not validate this with autophagy inhibitors, and further study is needed. Specifically, in the following studies, we will further improve the experiment to verify the mechanism of DHBF against CSVD.

## 5 Conclusion

**FIGURE 10:**
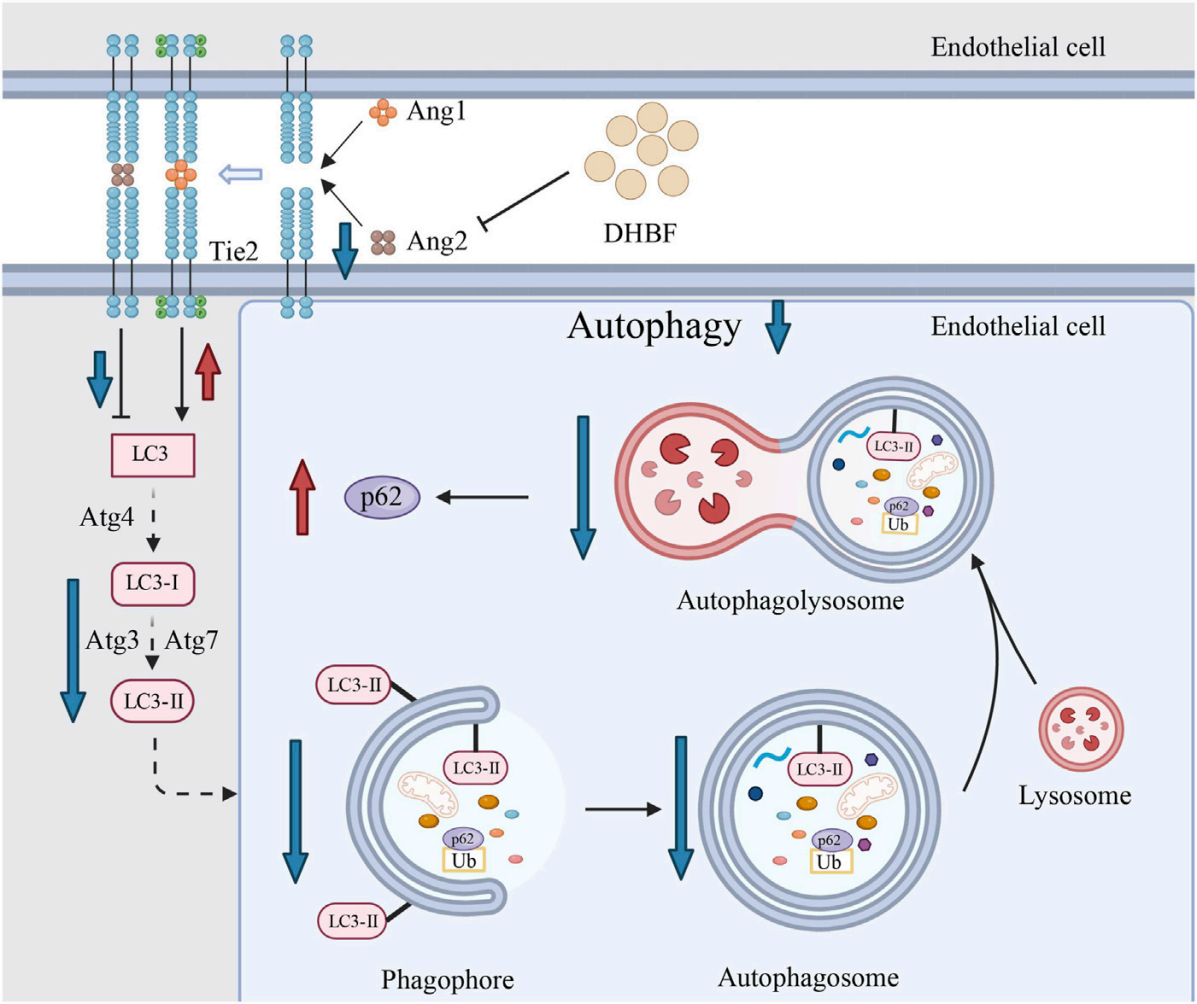
Schematic representation of the mechanism of action of DHBF. DHBF alleviates CSVD by inhibiting Ang2 and modulating the Angs-Tie2 pathway to prevent excessive autophagy in endothelial cells.

In this study, SHR rats with brain small vessel disease were used to validate the therapeutic effect of DHBF on CSVD. In addition, we constructed a mild hypoxic injury model of endothelial cells to simulate the condition of cerebral microvascular endothelial cells in patients with CSVD. Our findings verified that DHBF inhibited Ang2, enhanced cell activity, and promoted vascular stability through the Angs-Tie2 pathway. Furthermore, it inhibited excessive autophagy of endothelial cells, thus alleviating the injury of damaged endothelial cells (Figure 10). These results propose a potential effective target for CSVD and provide a new insight for subsequent drug development of CSVD.

## Data Availability

The original contributions presented in the study are included in the article/Supplementary Material, further inquiries can be directed to the corresponding authors.

## Abbreviations

CSVD, Cerebral small vessel disease; VCI, Vascular cognitive impairment; DHBF, The flavonoid of Dracocephalum heterophyllum Benth.; SHR, Spontaneously hypertensive rats; HUVECs, Human umbilical vein endothelial cells; Angs, Angiopoietins; Ang1, Angiopoietin-1; Ang2, Angiopoietin-2; Tie2, Tyrosine-protein kinase receptor 2; LC3, Microtubule-associated protein 1A/1B-light chain 3; p62, Polyubiquitin-binding protein p62; VEGF, Vascular endothelial growth factor; LSCI, Laser speckle contrast imaging; CBF, Cerebral blood flow; PAT, Photoacoustic tomography; GO, Gene ontology; BP, Biological processes; CC, Cellular components; MF, Molecular function; KEGG, Kyoto encyclopedia of genes and genomes; PPI, Protein-protein interaction; DC, Degree; LSCM, Laser scanning confocal microscopy; TEM, Transmission electron microscope; SaO2, Oxygen saturation; HbO, Oxyhemoglobin; HbR, Deoxyhemoglobin.
