# Supplementary material for: The flavonoid of Dracocephalum heterophyllum Benth. ameliorates cerebral small vessel disease by inhibiting the autophagy via Angs-Tie2 signaling pathway
Source: Front Pharmacol. 2025 May 13;16:1500307. doi: 10.3389/fphar.2025.1500307 (PMC12107102; doi:10.3389/fphar.2025.1500307)
Supplement: Supplementary file 1 [file DataSheet1.docx]

***Supplementary Material***

**Supplementary methods**

**Extraction of DHBF**

The plants were identified as *Dracocephalum heterophyllum* Benth. by researcher Guan-Mian Sheng of the Xinjiang Technical Institute of Physics and Chemistry in the Chinese Academy of Sciences. DHBF (20200102) was produced by pilot plant of Xinjiang Technical Institute of Physics and Chemistry Chinese Academy of Sciences. Briefly, the dried plants were crushed and. the powder was extracted by refluxing with 70% ethanol for 2 h, repeated 3 times. The solution was concentrated by rotary evaporation under vacuum at 50 ℃ and filtered to obtain crude extracts. The crude extracts were purified by AB-8 microporous resin column chromatography eluted with distilled water and 70% ethanol, respectively. The 70% ethanol eluted solution was collected and concentrated by rotary evaporation, followed by freezing dried under vacuum to obtained a final yellow powder.

**UPLC-Q-Orbitrap HRMS analysis**

The DHBF sample was analyzed using a UPLC-Q-Orbitrap HRMS system (Thermo Fisher Scientific, USA). Chromatographic separation was performed on an Accucord TM Vanquish C_18_ column (3 mm ×100 mm, 2.6 μm). The mobile phase was composed of 0.1% formic acid in water (A) and 0.1% formic acid in acetonitrile (B), and run under the following gradient program (Table S1). The flow rate was 400 μL/min and the column temperature were 30 ℃. The injection volume was 5 μL. The Q-Qrbitrap HRMS analysis was performed in both positive and negative ion modes on hybrid quadrupole-orbitrap high resolution mass spectrometry fitted with an ESI source. The collision energy (CE) was 40 ev, and the collision energy spread (CES) was 20 V. The parameters of the ESI source were as follows: the ion source voltage was 5,000 V for the positive ion mode and 4,000 V for the negative ion mode, and the source temperature (TEM) was 550 ℃. Ion source gas1 (GS1) and gas2 (GS2) were both set to 55 psi, and curtain gas (CUR) was set to 35 psi. Data was collected in Full MS/dd-MS^2^ mode over the m/z range of 100-1,000 and analyzed by Compound Discoverer 3.1 software.

Table S1 The parameters of gradient program.

| Time (min) | Flow (μL/min) | A% | B% |
| --- | --- | --- | --- |
| 0.0 | 400 | 95 | 5 |
| 3.5 | 400 | 85 | 15 |
| 6.0 | 400 | 70 | 30 |
| 6.5 | 400 | 70 | 30 |
| 12.0 | 400 | 30 | 70 |
| 12.5 | 400 | 30 | 70 |
| 18.0 | 400 | 0 | 100 |
| 22.0 | 400 | 0 | 100 |
| 25.0 | 400 | 0 | 100 |
| 26.0 | 400 | 95 | 5 |
| 30.0 | 400 | 95 | 5 |

**Animal experimental workflow diagram**

The workflow diagram for animal experiments is as follows:


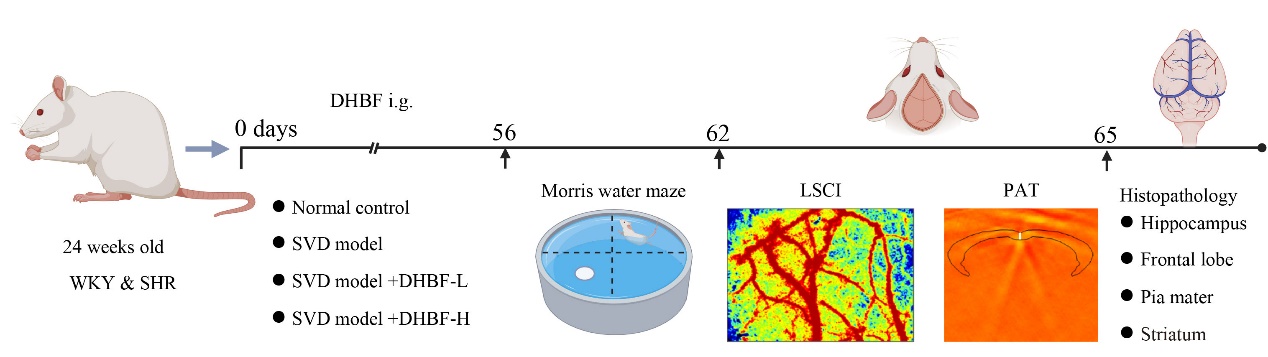


**Morris Water Maze Test**

A circular pool used in the training apparatus had a diameter of 150 cm. The pool was divided equally into four quadrants: Northeast (NE), Northwest (NW), Southeast (SE), and Southwest (SW), and was filled with water at 25℃. A white platform (12.5 cm in diameter) served as the platform, which was in the center of the SW quadrant and was submersed about 1 cm beneath the water surface. Rats were randomly placed into the water from the entry points of the four quadrants and allowed to find the platform (maximum swimming time was 60 seconds). The movement trajectory, escape latency, escape path length, and swimming speed were recorded.

**Photoacoustic Tomography**

The Nd: YAG-pumped optical parametric oscillator (Surelite, Continuum, California) system was used as a light source to illuminate 680-960 nm at a 20 Hz repetition rate. PA signals were detected by a 128-element concave transducer array with a central frequency of 5 MHz (Japan Probe Cooperation, Yokohama, Japan) after being transmitted to a data acquisition system for real-time imaging.

**GO and KEGG enrichment analysis and Protein-protein interaction (PPI) network analysis**

The compounds identified in DHBF were screened by pharmacokinetic ADME prediction using SwissADME (http://www.swissadme.ch/). Compounds labeled as having high Gastrointestinal absorption and matching 3 or more Druglikeness were used to predicted targets in SwissTargetPrediction (http://swisstargetprediction.ch/). And the CSVD-related targets were collected by searching the keyword “hypertension” and “cerebral small vessel disease” from GeneCards (https://www.genecards.org/). The intersection of active compounds and CSVD targets was obtained using Jvenn, (https://jvenn.toulouse.inrae.fr/app/example.html) and the intersection was submitted to the DAVID (https://david.ncifcrf.gov/) for Gene Ontology (GO) and Kyoto Encyclopedia of Genes and Genomes (KEGG) pathway enrichment analysis. Only enrichment results with p < 0.05 were considered statistically significant. The top 20 enrichment of KEGG analysis and GO analysis including biological processes (BP), cellular component (CC), molecular function (MF) were visualized as a bar graph by SRplot (<https://www.bioinformatics.com.cn/srplot>).

PPI analysis was performed by submitting the intersection targets to the STRING (https://cn.string-db.org/). The species was limited to Homo sapiens and the Minimum required interaction score was set to 0.9, the disconnected nodes in the network were hidden. Subsequently, the PPI results were exported from STRING and imported into Cytoscape 3.9.1 for analyzing and visualizing. To screen out the key targets for DHBF treatment of CSVD, the degree centrality (DC) and betweenness centrality (BC) of network nodes were analyzed using cytoscape 3.9.1.

**Scratch wound healing assay**

Exponentially growing HUVECs were seeded into 24-well plates. The wounds were made by a sterile 10 μL pipette tip through a pre-marked line. After clearing the debris from the line scratches, cells were rinsed three times with PBS followed by treated cells under different conditions for 24 h. HUVECs were photographed at 0 h and 24 h post-wounding and the scratch area was calculated by ImageJ 1.5 for data analysis.

**Cell migration and invasion assays**

The HUVECs were diluted to a suitable concentration with serum-free medium and 100 μL cell suspension was added to the upper chamber of the transwell 24-well plate (without Matrigel for the Cell migration assay) or the chamber with Matrigel (for the Cell invasion assay), while 600 μL complete medium was added to the lower chamber. The 24-well plate with transwell chamber was incubated at 37℃ for 24 hours. The cells that traversed to the lower chamber were fixed using 4% methanol for 20 min and stained with Giemas dye. Subsequently, a cotton swab was used to remove the cells on the surface of the membrane, then the colored cells were photographed and counted using a microscope.

**Quantitative real‐time polymerase chain reaction (qRT‐PCR)**

Total RNA samples from HUVECs were extracted using the TRIzol™ Reagent (ambion, USA) according to the manufacturer’s instructions. The complementary DNA (cDNA) was synthesized from 800 ng of total RNA by using a 5× All-In-One RT MasterMix with AccuRT Genomic DNA Removal Kit (abm, CAN). Then qRT-PCR analysis was performed with EvaGreen Express 2×qPCR MasterMix-Low Rox kit (abm, CAN) on real-time PCR instrument. (ABI, USA). The Primers are shown in Table S2. The target gene expression level relative to the endogenous control gene (GAPDH) was calculated by the 2^-∆∆CT^ method.

Table S2. the primers used for quantitative real-time PCR.

| Gene | Primer sequences 5’→3’ |
| --- | --- |
| Foxo1 | Forward: AGTGGATGGTCAAGAGCGTG |
|  | Reverse: TGTTGTCCATGGATGCAGCT |
| Lc3b | Forward: GCATCCAACCAAAATCCCGG |
|  | Reverse: TCACCAACAGGAAGAAGGCC |
| P62 | Forward: ACAGGTGAACTCCAGTCCCT |
|  | Reverse: CAGCCGCCTTCATCAGAGAA |
| Ang1 | Forward: GCCTGATCTTACACGGTGCT |
|  | Reverse: CGTAAGGAGTAACTGGGCCC |
| Ang2 | Forward: AATGCTAACAGGAGGCTGGT |
|  | Reverse: GGATCATCATGGTTGTGGCC |
| Tie2 | Forward: CCTTGGCTCTGCTGGAATGA |
|  | Reverse: CACGTTTTGGAAGGCTTGGG |
| Gapdh | Forward: TGTTGCCATCAATGACCCCTT |
|  | Reverse: CTCCACGACGTACTCAGCG |

**Western blot**

Total proteins from different groups were isolated using RIPA buffer supplemented with PMSF, and protease inhibitors (Boster Biological Technology Co., Ltd., AR0105). The concentrations of proteins were detected by the Easy II Protein Quantitative Kit (TransGen Biotech Co., Ltd., China). Proteins were separated by 10% SDS-PAGE and electrotransferred onto PVDF membrane. The membranes were blocked with 5% non-fat milk for 1 h at temperature. Then the membranes were incubated overnight at 4℃ with primary antibodies against FoxO1 (1:400, Boster, PB0191), p62 (1:400, Boster, BA2849), Ang1 (1:500, Boster, PB0092), Ang2 (1:500, Boster, A00370), TIE2 (1:400, Boster, A01274-1), LC3B (1:400, Boster, BM4827) and β-actin (1:1000, Sino Biological, 100166-MM10). After washing three times with TBST, the membranes were incubated with secondary antibodies (Goat anti-Mouse IgG H&L for β-actin and Goat anti-Rabbit IgG H&L for other genes) for 1 h at room temperature, followed by washing three times with TBST. Subsequently, the chemiluminescence imager system was used for detecting the membranes, and β-actin served as the control protein to quantify the expression of related proteins.

**Molecular docking**

Firstly, download the molecular structure of active ingredients and the protein crystal structure of Ang2 from Pubchem and Protein Data Bank (<http://www.rcsb.org/>), respectively. Then the protein structure was pretreated by Schrodinger Maestro, including the removal of water molecules, addition with hydrogens, and so on. Secondly, the mating pockets were predicted by Schrodinger Maestro. Then molecular docking was also performed using the Schrodinger Maestro. The docking result with the lowest binding energy was selected for analysis. The target has a certain binding activity with the active ingredients if the binding energy is less than -5 KJ/mol. The best docking model was visualized by Schrodinger Maestro.

**Supplementary figure**


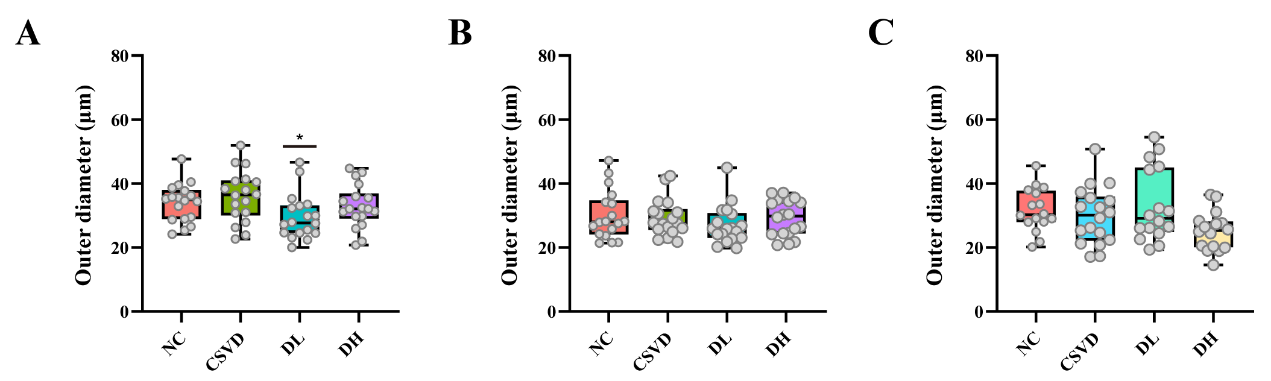


**Supplementary figure 1.** The effects of DHBF on outer diameter of cerebral small vessel in CSVD rats. Statistical analysis of outer diameter of (A) pia mater, (B) frontal lobe and (C) striatum.


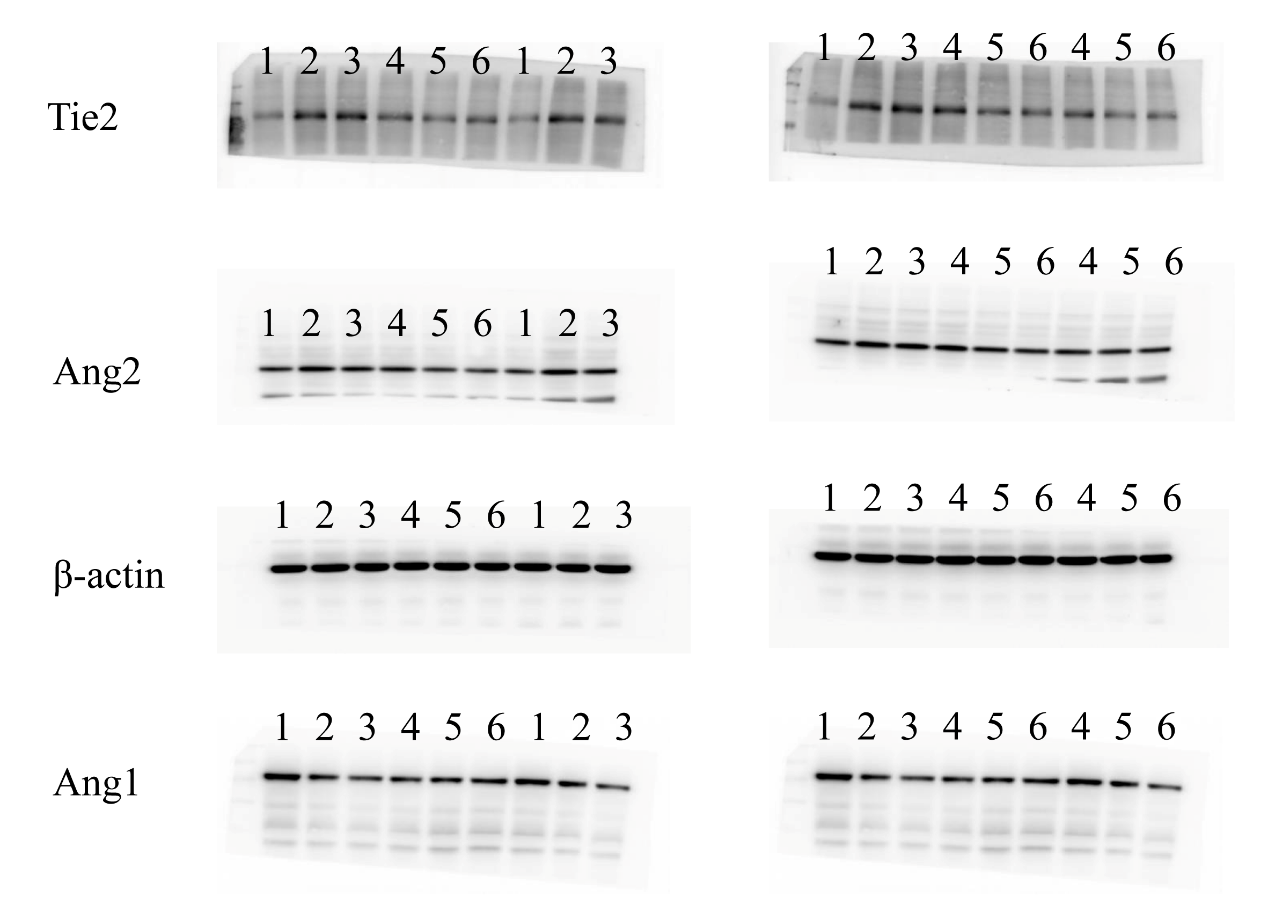


**Supplementary figure 2.** **Effects of DHBF on Angs-Tie2 pathway.** The protein in HUVECs from Control group (No.1), Model group (No.2), Hypoxia+10 mg/mL DHBF group (No.3), Hypoxia+25 mg/mL DHBF group (No.4), Hypoxia+50 mg/mL DHBF group (No.5), Positive group (No.6).


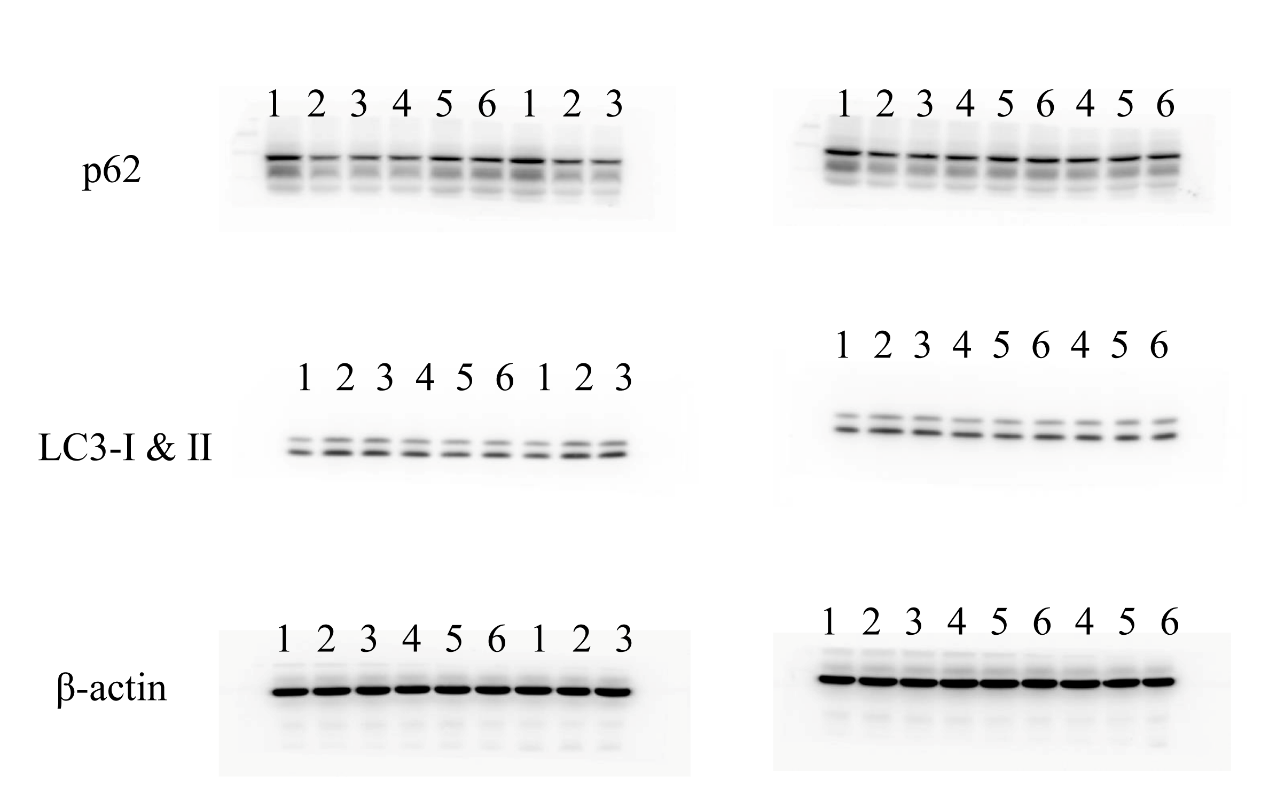


**Supplementary figure 3.** **Effects of DHBF on autophagy in hypoxia HUVECs.** The protein in HUVECs from Control group (No.1), Model group (No.2), Hypoxia+10 mg/mL DHBF group (No.3), Hypoxia+25 mg/mL DHBF group (No.4), Hypoxia+50 mg/mL DHBF group (No.5), Positive group (No.6).


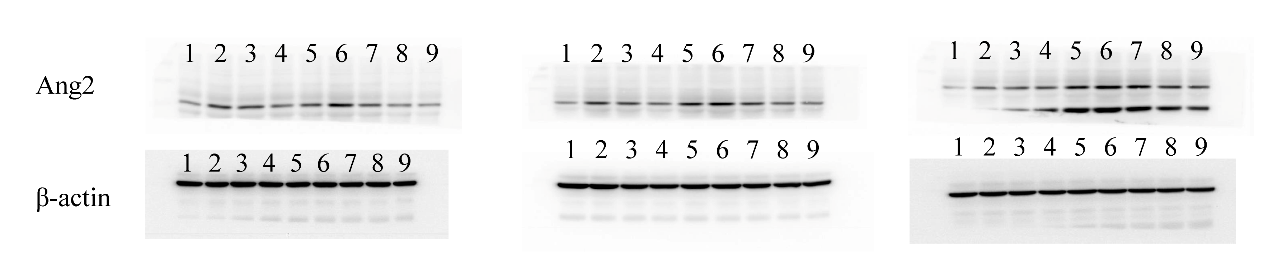


**Supplementary figure 4.** **The role of Ang2 on proliferation, migration and invasion ability of hypoxia HUVECs.** The protein in HUVECs from Control group (No.1), Model group (No.2), sh-NC group (No.3), sh-Ang2 group (No.4), over-NC group (No.5), over-Ang2 group (No.6), Hypoxia+10 mg/mL DHBF group (No.7), Hypoxia+25 mg/mL DHBF group (No.8), Hypoxia+50 mg/mL DHBF group (No.9)
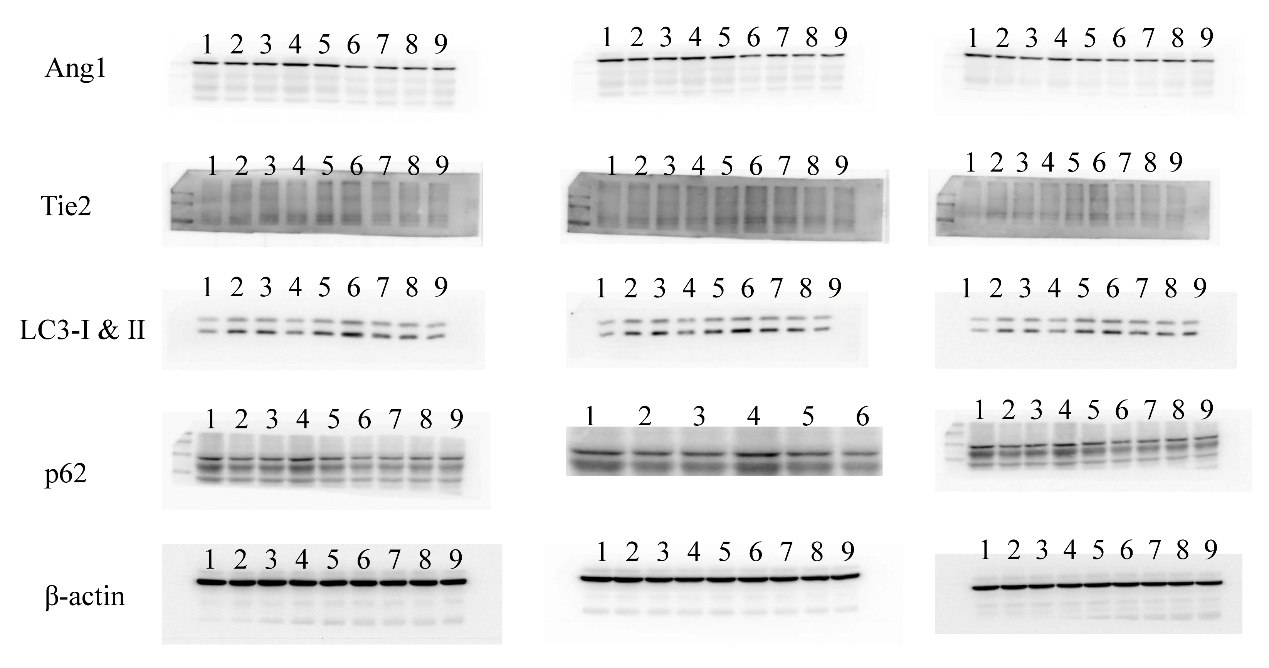


**Supplementary figure 5.** **The role of Ang2 on Angs-Tie2 pathway and autophagy of hypoxia HUVECs**. The protein in HUVECs from Control group (No.1), Model group (No.2), sh-NC group (No.3), sh-Ang2 group (No.4), over-NC group (No.5), over-Ang2 group (No.6), Hypoxia+10 mg/mL DHBF group (No.7), Hypoxia+25 mg/mL DHBF group (No.8), Hypoxia+50 mg/mL DHBF group (No.9).


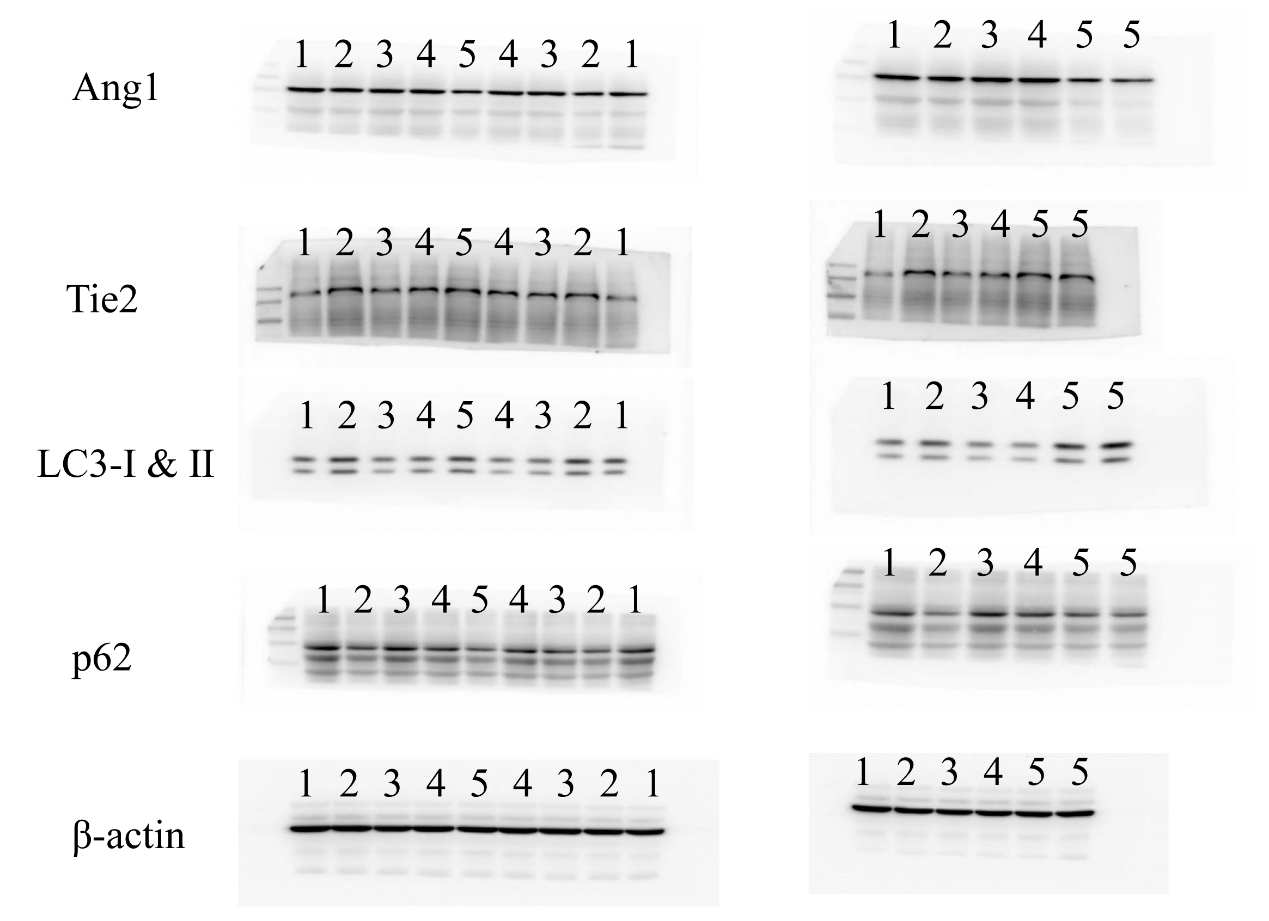


**Supplementary figure 6. DHBF inhibited autophagy and regulated Ang1-Tie2 pathway in hypoxia HUVECs via targeting downregulation of Ang2.** The protein in HUVECs from Control group (No.1), Model group (No.2), Hypoxia+50 mg/mL group (No.3), Hypoxia+over-NC group (No.4), Hypoxia+over-Ang2 group (No.5).

.

Table S3. Chemical constituents identified in DHBF consistent with oral availability, druglikeness, and blood-brain barrier based on UPLC-Q-Orbitrap HRMS

| NO. | Compound | | RT (min) | | Molecular Weight | | Formula | | fragment | Database Search Score | |
| --- | --- | --- | --- | --- | --- | --- | --- | --- | --- | --- | --- |
| 1 | Erucamide | | 21.52 | | 337.33 | | C_22_H_43_NO | | 338, 339, 321, 379 | 96.5 | |
| 2 | Carvone | | 6.16 | | 150.11 | | C_10_H_14_O | | 151, 123, 109 | 92.3 | |
| 3 | Loliolide | | 6.75 | | 196.11 | | C_11_H_16_O_3_ | | 197, 179, 133, 107 | 93.3 | |
| 4 | Vanillin | 5.90 | | 152.05 | | C_8_H_8_O_3_ | | 153, 125, 111, 93, 65 | | | 85.4 |
| 5 | (-)-caryophyllene oxide | 9.65 | | 220.18 | | C_15_H_24_O | | 221, 203, 147, 107 | | | 91.1 |
